# Uncovering the complex genetics of human character

**DOI:** 10.1038/s41380-018-0263-6

**Published:** 2018-10-03

**Authors:** Igor Zwir, Javier Arnedo, Coral Del-Val, Laura Pulkki-Råback, Bettina Konte, Sarah S. Yang, Rocio Romero-Zaliz, Mirka Hintsanen, Kevin M. Cloninger, Danilo Garcia, Dragan M. Svrakic, Sandor Rozsa, Maribel Martinez, Leo-Pekka Lyytikäinen, Ina Giegling, Mika Kähönen, Helena Hernandez-Cuervo, Ilkka Seppälä, Emma Raitoharju, Gabriel A. de Erausquin, Olli Raitakari, Dan Rujescu, Teodor T. Postolache, Joohon Sung, Liisa Keltikangas-Järvinen, Terho Lehtimäki, C. Robert Cloninger

**Affiliations:** 1 Department of Psychiatry, Washington University School of Medicine, St. Louis, MO USA; 2 Department of Computer Science, University of Granada, Granada, Spain; 3 Department of Psychology and Logopedics, University of Helsinki, Helsinki, Finland; 4 Department of Psychiatry, Martin-Luther-University Halle-Wittenberg, Halle, Germany; 5 Department of Epidemiology, School of Public Health and Institute of Health and Environment, Seoul National University, Seoul, Korea; 6 University of Oulu, Unit of Psychology, Faculty of Education, Oulu, Finland; 7 Anthropedia Foundation, St. Louis, MO USA; 8 Department of Psychology, University of Gothenburg, Gothenburg, Sweden; 9 Blekinge Centre of Competence, Blekinge County Council, Karlskrona, Sweden; 10 Department of Clinical Chemistry, Fimlab Laboratories, Faculty of Medicine and Life Sciences, Finnish Cardiovascular Research Center-Tampere, University of Tampere, Tampere, Finland; 11 Ludwig-Maximilian University, University Clinic, Munich, Germany; 12 Department of Clinical Physiology, Tampere University Hospital and Faculty of Medicine and Life Sciences, University of Tampere, Tampere, Finland; 13 Departments of Psychiatry and Neurosurgery, University of South Florida, Tampa, FL USA; 14 Institute of Neurosciences, Department of Psychiatry and Neurology, School of Medicine, University of Texas Rio-Grande Valley, Harlingen, TX USA; 15 Research Centre of Applied and Preventive Cardiovascular Medicine, Department of Clinical Physiology and Nuclear Medicine, Turku University Hospital, University of Turku, Turku, Finland; 16 Department of Psychiatry, School of Medicine, University of Maryland, Baltimore, MD USA; 17 Rocky Mountain Mental Illness, Research, Education, Clinical Center for Veteran Suicide Prevention, Denver, CO USA; 18 Department of Genetics, Department of Psychological and Brain Sciences, and School of Medicine, School of Arts and Sciences, Washington University, St. Louis, MO USA

**Keywords:** Diagnostic markers, Genetics, Neuroscience, Psychology

## Abstract

Human personality is 30–60% heritable according to twin and adoption studies. Hundreds of genetic variants are expected to influence its complex development, but few have been identified. We used a machine learning method for genome-wide association studies (GWAS) to uncover complex genotypic–phenotypic networks and environmental interactions. The Temperament and Character Inventory (TCI) measured the self-regulatory components of personality critical for health (i.e., the character traits of self-directedness, cooperativeness, and self-transcendence). In a discovery sample of 2149 healthy Finns, we identified sets of single-nucleotide polymorphisms (SNPs) that cluster within particular individuals (i.e., SNP sets) regardless of phenotype. Second, we identified five clusters of people with distinct profiles of character traits regardless of genotype. Third, we found 42 SNP sets that identified 727 gene loci and were significantly associated with one or more of the character profiles. Each character profile was related to different SNP sets with distinct molecular processes and neuronal functions. Environmental influences measured in childhood and adulthood had small but significant effects. We confirmed the replicability of 95% of the 42 SNP sets in healthy Korean and German samples, as well as their associations with character. The identified SNPs explained nearly all the heritability expected for character in each sample (50 to 58%). We conclude that self-regulatory personality traits are strongly influenced by organized interactions among more than 700 genes despite variable cultures and environments. These gene sets modulate specific molecular processes in brain for intentional goal-setting, self-reflection, empathy, and episodic learning and memory.

## Introduction

Strong evidence for substantial heritability of human personality comes from family, twin, and adoption studies [1]. However, the genetic and phenotypic architecture of human personality is complex and has remained uncertain despite recent advances in genomics and phenomics [2–4]. In general, geneticists must expect the likelihood that many genes affect each trait and each gene affects many traits [5]. When the architecture is complex, the same genetic networks may lead to different phenotypic outcomes (a phenomenon called multifinality in development or pleiotropy in genetics) [6–8]. Likewise, different genetic networks in complex systems may lead to the same outcome (equifinality, which is also described as heterogeneity) [8, 9].

Human personality is a striking example of the challenges involved in identifying the specific genes and molecular processes that influence complex traits. Twin studies indicate that between 30% and 60% of the phenotypic variance in personality, as assessed by a variety of instruments, is genetic in origin [10–14]. However, adoption studies and studies that include other family members along with twins show that most of the heritability of personality, as assessed by a variety of instruments, is likely to depend on complex interactions among multiple gene loci (i.e., epistasis) or multiple alleles at a locus (i.e., dominance), rather than the average effects of individual genes [11, 13–17]. Put another way, many genes are likely to operate in concert, not separately, to influence the heritability and development of personality. Nevertheless, despite extensive past effort, genome-wide association studies (GWAS) of personality have found few significant associations using a variety of personality instruments [18–20]. The frequent failure to account for most of the heritability of complex traits has been called the “missing” [21] or “hidden” [22] heritability problem.

The Temperament and Character Inventory (TCI) measures two domains of personality hypothesized to be related to different genetic and neuronal networks [23]. Imaging studies show that TCI character traits are associated with brain networks for intentional and meta-cognitive processes, such as self-reflection, goal-setting, empathy, and episodic learning, whereas temperament traits are related to generating and conditioning automatic behaviors, such as stress reactions [24–28]. In this article, we focus on TCI character traits of self-directedness (i.e., purposeful, responsible vs. aimless, blaming), Cooperativeness (i.e., helpful, empathic vs. hostile, self-centered), and self-transcendence (i.e., altruistic, spiritual vs. individualistic, skeptical). These are the self-regulatory components of personality that determine the degree to which a person's adaptive functioning is healthy or unhealthy [29]. In related articles, we examine temperament traits and their relations with character in the same samples.

We have chosen to apply strictly data-driven machine learning methods in a person-centered approach to GWAS to uncover the complex genotypic and phenotypic architecture of personality [6, 30, 31] (Supplementary Figure S1). We postulate that personality heritability is not missing, but is distributed in multiple networks of interacting genetic and environmental variables that influence different people [6, 31–33].

## Subjects and methods

### Description of the samples

Our discovery sample was the Young Finns Study, an epidemiological study of 2149 healthy Finnish children followed regularly from 1980 (ages, 3–18 years) to 2012 (ages, 35–50 years) [34]. Childhood environments were directly assessed with the rearing parents in 1980 and 1983 [35–39]. Adult environments and life events were assessed with subjects in 2001 [40, 41]. All subjects (56% women) had thorough standardized genotypic and phenotypic assessments, including administration of the TCI in 1997, 2001, 2007, and 2012 [34, 42].

We replicated the results in two independent samples of healthy adults from Germany [43, 44] and Korea [45, 46], in which comparable genotypic and phenotypic features were available (see Supplement). The Korean study involved 1052 unrelated individuals extracted from a national register (aged 28–81, 57% women). The German study involved 902 subjects (aged 20–74, 49% women) randomly selected from Munich city registry and screened to exclude anyone with a history of psychiatric illness in themselves or their first-degree relatives.

### Personality assessment

All subjects completed the TCI to assess seven heritable dimensions of personality [23, 47]. The TCI measures four dimensions of temperament and three dimensions of character (self-directedness, cooperativeness, and self-transcendence) with strong reliability, as described in Supplementary Section 1 and Supplementary Table S1 [23, 47]. The 13 subscales of character from the TCI were used as the primary data about character in all three samples (Supplementary Section 2). Character profiles for each person were based on median splits of each subscale to distinguish high and low scorers [48].

### Personality health indices

People at risk of unhealthy personality were identified as the bottom decile of the sum of TCI self-directedness and cooperativeness [48]. Prior work shows this criterion indicates ill-being or personality disorder (i.e., poor physical, mental, and social functioning) [49, 50]. In contrast, people with healthy personalities were identified as the top decile of the product of all three TCI character traits. Prior work shows that this criterion indicates well-being or flourishing (i.e., superior physical, mental, and social functioning) [29, 48, 51]. These indices provided consistent measures of the health status of subjects in all three samples. The health value of a set (i.e., group of people) is the average value of its members.

We also identified an empirical index of character functioning by clustering the 13 character subscales of the TCI (Supplementary Section 3 and Table S2). The empirical index of character provided a single comprehensive measure of character functioning that could be associated a posteriori with each SNP set based on semi-supervised learning [52] and used in SNP-set Kernel Association Test (SKAT) [32, 33] and heritability analyses. It was highly correlated with the other health indicators (*p* < E-20, RMSE 0.03).

### Genotyping

The Finnish sample was genotyped by using Illumina Human670-Quad Custom, (i.e., Illumina 670k custom) arrays [53]. The Korean sample used Affymetrix Genome-Wide Human SNP Array 6.0 and Illumina HumanCore [45]. The German sample used Affymetrix Genome-Wide Human SNP Array 6.0, Illumina OMNI Express and the 300 Array, prephased and imputed with SHAPEIT2 and IMPUTE2. Some German individuals had also been genotyped on Illumina Omni1-Quad. Quality control was performed for all samples as in prior work [6] (Supplementary Section 3).

After quality checks, a subset of SNPs were preselected with the PLINK software suite [54] to reduce the large search space using a generously inclusive threshold (*p*-value <0.01 without Bonferroni correction) for possible association with character, taking gender and ethnicity into account as covariates of the individual SNPs. Preselecting SNPs identified SNPs that have weak associations with character that are not individually significant genome wide after Bonferroni correction, but provided presumptive candidates for epistatic interactions in a SNP set. The preselection also identified SNPs with a strong additive effect individually, thereby providing a manageably sized initial pool of SNPs as candidates for both the additive and non-additive components of the genetic architecture of character. We accounted for ethnicity in each sample by using the first three principal components for ancestral stratification of SNP genotypes (Supplementary Section 3) [55].

### Computational procedures

The cluster analyses used the validated Generalized Factorization Method, which utilizes deep non-negative matrix factorization (NMF) to uncover naturally occurring (i.e., unsupervised) associations between patterns across different types of data, including genetics [56–59] and neuroimages [30, 60]. The clustering was entirely data driven without restrictive assumptions about the number or content of the clusters [31]. For example, clusters may have different features, and one subject can belong to more than one cluster [6, 30, 31, 56, 61]. The recurrent application of the clustering process is summarized and schematically related to unsupervised deep NMF learning in Supplementary Figure S1 [62]. The advantages of this clustering approach over alternative analyses of single or multiple markers are described in Supplementary Section 4.

Our web server application for phenotype–genotype many-to-many relations analysis (PGMRA) in GWAS is published [31] and online at http://phop.ugr.es/fenogeno. The PGMRA method and algorithm are also summarized in Supplementary Sections 5 and 6, which includes a semi-supervised classifier of phenotypes from genotypes. PGMRA properly accounts for linkage disequilibrium (LD) efficiently (i.e., without loss of information about complex genotypic–phenotypic relations) (Supplementary Section 4). Statistical analysis correcting for multiple comparisons, as well as gender and ethnicity as covariates of the SNP sets, was performed by the SKAT [32, 33], also accessible via PGMRA. Heritability was estimated from a trimmed regression of SNPs on the empirical index of character controlling for outliers and environmental variables [63, 64] (also see Supplementary Section 7).

Replicability of results was evaluated in the three independent samples for SNP sets, phenotypic sets, and genotypic–phenotypic relations using multi-objective optimization techniques [6], as detailed in Supplementary Section 8. We also evaluated how well the individual genotypic sets were able to predict the classification of the phenotypes in each sample using the PGMRA classifier (Supplementary Section 9). Further details are available in Supplementary Information and elsewhere [56–59].

## Results

### Identifying SNP sets as candidates for causal variability

**Fig. 1:**
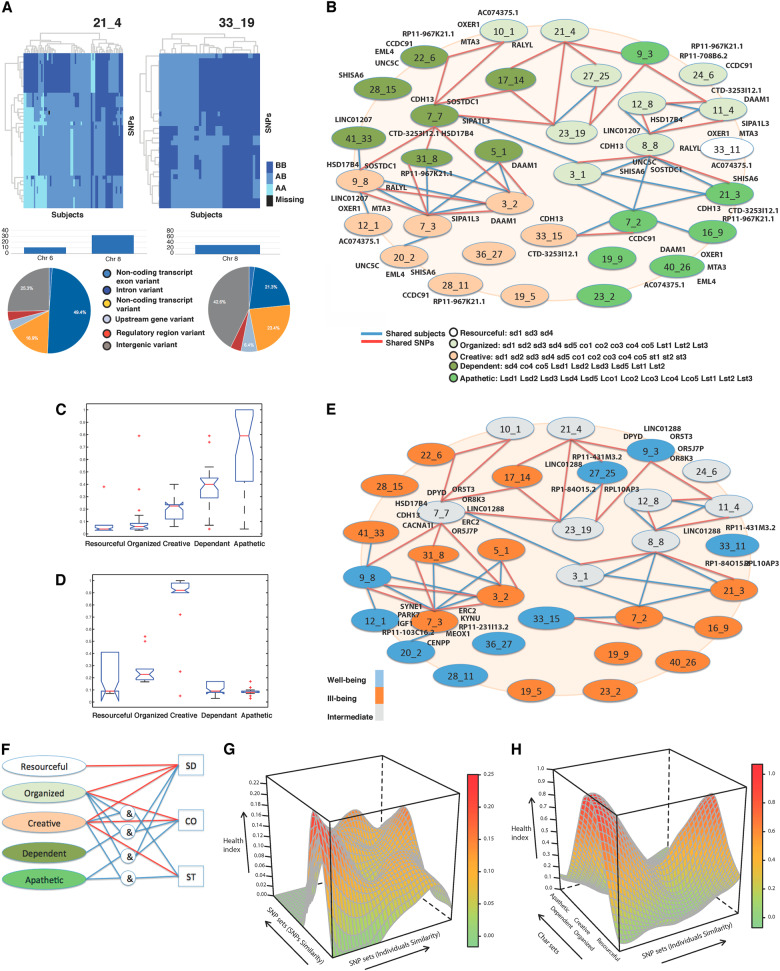
**a** Two examples of SNP sets are represented as heatmap submatrices or biclusters. SNP sets were identified by distinct patterns of molecular features of SNPs in subgroups of subjects. Allele values are indicated as BB (dark blue), AB (intermediate blue), AA (light blue), and missing (black). SNP sets were labeled for specificity by a pair of numbers representing the maximum number of clusters from which the bicluster was selected (e.g., 33 clusters may produce more specific than 21) and the order in which they were selected by the method (e.g., 4th bicluster or factor selected by FNMF when the maximum number of clusters was 21) and usually have a prefix G for genotype or P for phenotype. Only a subset of optimal and cohesive sets are selected across all number of clusters (See Supplementary Methods). The SNPs within each SNP set can map to different chromosomes (e.g., 6 and 8) and exhibit distinct molecular consequences (see Supplementary Table S3). The pie chart shows the percentage of SNPs within a SNP set that belong to each type of consequence. **b** Dissection of a GWAS in a Finnish population to identify the genotypic and phenotypic architecture of personality measured by the TCI. The genotypic network is depicted as nodes (SNP sets) linked by shared SNPs (blue lines) and/or subjects (red lines) (see also Supplementary Figure S3A for additional subnetworks). Each SNP set maps to one or more genes (see Supplementary Table S6 for full list of genes associated with each SNP set). SNP sets associated with each of the five general character profiles are distinguished by color-coding as shown in the legend (see Table 3). **c**, **d** Comparison of level of ill-being (**c** where high values indicate ill-being) and for level of well-being (**d** where high values indicate well-being) in groups of subjects with each of the five character profiles specified by both phenotypic and genotypic information (evaluated by ANOVA). (Compare with either genetic or phenotypic assessment alone in Supplementary Figure S6). **e** Variation in health status of SNP sets: well (blue, see **d**), ill (orange, see **c**), intermediate (gray). **f** 12 genotypic-phenotypic pipelines connect different sets of genes to the same character dimension (see also Supplementary Tables S9–S12). Red lines indicate direct connections, whereas blue lines and “&” indicate composite connections. **g** Surface showing the pattern of health status of the subjects in this study based on SNP set information only (i.e., interpolation from Table 1). The probability of well-being in the z-axis varies from high (red for high well-being) to low (green). The order of the SNP sets is based on shared subjects (*x*-axis) and on shared SNPs (*y*-axis) measured by hypergeometric statistics, so SNP sets sharing more SNPs and/or subjects are nearby (see ill health surface in Supplementary Figure S4). **h** Surface showing the pattern of health status of subjects based on both genotypic information (SNP sets) and phenotypic information (character sets) (as in Table 3). The probability of well-being in the *z*-axis varies from high (red, high well-being) to low (green). The sharing of subjects is shown for both SNP sets (*x*-axis) and character sets (*y*-axis) (see ill health surface in Supplementary Figure S5)

**Fig. 2:**
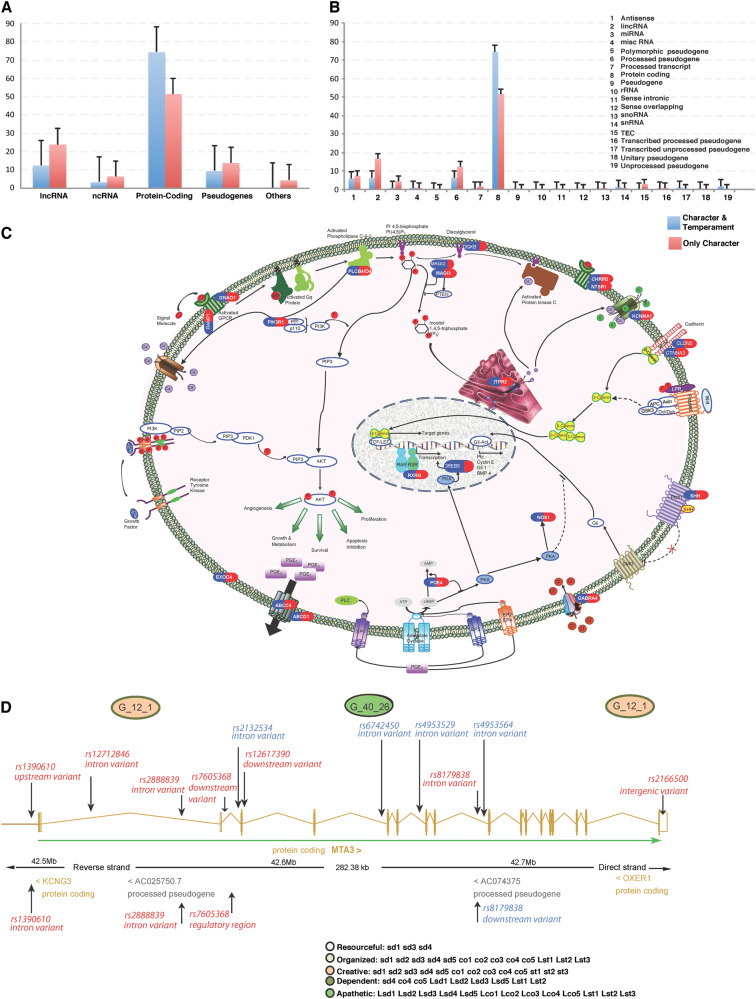
**a**, **b** Types of genetic variants mapped by SNP sets associated with character: **a** Specific molecular consequences (Supplementary Table S5) and **b** their subtypes. Genes related only to character sets (red) were less often protein coding and more often RNA genes than those also associated with temperament sets (blue color). **c** Cell displaying the molecular pathways containing genes associated only with the organized profile. The uncovered genes influence the phosphatidyl inositol/calcium second-messenger signaling system that regulates the seeking of food and other goals in response to external environmental signals (see also Supplementary Tables S4, S7). **d** Multiple SNPs within a SNP set can affect a single or multiple genes in many ways (Supplementary Table S3). Within the MTA3 gene, SNPs in the SNP set G_12_1 may affect both coding and regulatory regions (thereby inhibiting transcription), whereas SNPs from SNP set 40_26 are mostly located in intronic regions (thereby blocking or decreasing protein production). The SNP sets are associated with profiles exhibiting distinct character features (creative vs. apathetic)

We exhaustively identified 902 non-identical but possibly overlapping SNP sets in the Finnish sample using PGMRA without knowledge of the phenotype. The SNP sets were comprised of different numbers of SNPs and/or subjects, regardless of their phenotypic status. The SNPs were mapped to diverse functional classes of genetic variants that may be located on different chromosomes, frequently even within a single SNP set (Figs. 1a, 2a–d). SNP sets are organized as networks of multilocus genotypes (Fig. 1a, b; Supplementary Figure S2, Supplementary Table S3). They were labeled by a genotypic identification ‘G’, followed by two numbers: the first indicates the maximum number of clusters and the second indicates the order of selection by the algorithm. SNP sets were associated with different health risks (Table 1, Supplementary Table S2).

**Table 1:** Description of 42 SNP sets associated with character sets (*p* < 1E-05)

Finnish sample								Probability of health		^a^Genes
SNP sets	SNP-set name	% Coding	SKAT *p*-value	Best SNP	Average SNPs	^a^Subjects	^a^SNPs	Well-being	Ill-being	
G_3_1	Inositol-calcium signaling*	62	2.88E-102	3.01E-05	2.64E-01	311	2163	0.06	0.1	>300
G_8_8	Inositol/chemokine pathways	67	2.21E-55	8.55E-05	1.99E-01	224	611	0.08	0.07	291
G_7_2	GPCR dysregulation	62	2.07E-31	9.00E-05	2.44E-01	211	303	0.09	0.23	142
G_7_3	Neurogenesis	63	1.67E-20	1.07E-04	1.85E-01	133	364	0.17	0.36	136
G_11_4	Inositol signaling	55	1.22E-19	9.00E-05	3.29E-02	141	172	0.07	0.04	51
G_12_8	Neuroprotection	62	1.49E-16	2.53E-04	3.39E-01	173	285	0.09	0.03	111
G_7_7	Olfaction	56	1.32E-11	3.27E-05	2.25E-01	145	193	0.03	0.1	55
G_36_29	Electron transport	50	5.37E-09	4.27E-04	3.37E-01	25	185	0.08	0.48	76
G_31_8	Neurotrophin	55	1.15E-08	2.90E-05	3.13E-01	54	183	0.09	0.54	64
G_28_15	Histone methylation	44	2.77E-08	3.76E-05	2.23E-01	101	123	0.08	0.38	34
G_9_8	Neuroregulation	57	3.82E-08	1.11E-04	3.48E-01	209	230	0.17	0.12	77
G_24_6	GFI1-neurite outgrowth	36	5.22E-08	2.65E-04	7.47E-02	72	63	0.1	0.08	14
G_19_5	DARPP320-neuroplasticity	30	8.31E-08	1.23E-04	2.13E-01	86	59	0.16	0.22	10
G_33_15	ERK-neurodevelopment	53	1.60E-07	4.47E-04	8.47E-02	26	67	0.27	0.23	19
G_23_2	Biogenic amine synthesis	50	2.24E-07	1.39E-04	9.46E-02	42	56	0.05	0.29	8
G_3_2	PAK-neuroprotection	63	3.08E-07	1.70E-05	1.80E-01	133	197	0.18	0.24	35
G_22_6	Blood–brain barrier	59	3.37E-07	2.53E-04	2.35E-01	37	93	0.08	0.16	32
G_34_13	CREB-episodic learning	54	3.88E-07	2.38E-04	1.28E-01	41	49	0.05	0.29	13
G_40_26	Dopamine-feedback	65	6.08E-07	3.88E-04	2.61E-01	39	98	0.08	0.36	17
G_21_3	Cellular senescence	62	1.12E-06	1.85E-04	3.55E-01	60	117	0.1	0.23	34
G_20_2	Enhanced memory	79	1.59E-06	2.78E-04	2.34E-01	25	80	0.24	0.12	19
G_28_11	Sensory transduction	44	2.07E-06	8.29E-04	2.71E-01	32	81	0.22	0.06	9
G_12_1	Episodic learning	61	5.06E-06	9.00E-05	3.41E-01	146	189	0.2	0.06	66
G_41_33	GPCR neuroplasticity	40	5.31E-06	4.91E-04	2.71E-01	56	76	0.11	0.21	15
G_9_3	Pyrimidine metabolism	50	6.03E-06	4.54E-05	2.55E-02	164	35	0.12	0.04	6
G_26_14	Glucose transport	59	6.98E-06	1.08E-04	2.20E-01	46	75	0.09	0.24	27
G_20_3	Fatty acid oxidation	48	8.03E-06	2.07E-04	3.04E-01	36	82	0.03	0.33	21
G_23_19	Org2-RNA	0	1.17E-05	3.76E-05	9.37E-04	87	32	0.08	0.03	4
G_17_14	Dep-RNA	0	1.17E-05	3.76E-05	9.37E-04	47	32	0.09	0.15	4
G_27_25	Org3-RNA	0	1.17E-05	3.76E-05	9.37E-04	54	32	0.11	0.07	4
G_41_40	Apath-RNA	0	1.17E-05	3.76E-05	9.37E-04	34	32	0.09	0.03	4
G_38_10	Org5-RNA	0	1.17E-05	3.76E-05	9.37E-04	27	32	0.15	0.19	4
G_33_19	Res-RNA	0	1.17E-05	3.76E-05	9.37E-04	43	32	0.09	0.12	4
G_21_4	Org1-RNA	0	1.28E-05	3.76E-05	9.41E-02	68	43	0.07	0.07	4
G_10_1	Learning/memory	47	1.29E-05	3.27E-05	7.44E-02	131	48	0.07	0.06	15
G_35_12	Org4-RNA	0	1.33E-05	3.76E-05	9.32E-04	45	31	0.16	0.09	3
G_35_4	O-linked glycosylation	57	1.39E-05	3.74E-04	2.83E-01	42	37	0.05	0.24	7
G_5_1	CDK neuroplasticity	100	1.78E-05	1.70E-05	5.60E-02	100	91	0.17	0.4	1
G_19_9	Aurora-B	38	1.91E-05	2.65E-04	1.25E-01	20	46	0.1	0.4	8
G_36_27	Aging regulation	25	1.96E-05	8.29E-04	1.60E-01	27	57	0.33	0.22	4
G_16_9	Olfactory signaling	58	2.00E-05	2.31E-04	1.79E-01	64	36	0.08	0.17	12
G_33_11	Self-control	50	3.67E-05	2.65E-04	9.39E-02	43	32	0.26	0.07	15

The SNP sets are named based on molecular pathways and neuronal functions of the genes that distinguish the sets from one another (see Supplementary Table S4). Percentage coding indicates the percentage of protein coding genes. Strengths of association are compared for the SNP set, the best SNP, and average SNP based on SKAT *p*-values. The number of subjects and SNPs comprising each SNP set is specified. The probabilities of the well-being and ill-being are given for subjects in each SNP set (see also Supplementary Table S2)

^a^Genes indicates the genes mapped by the SNP set (Figure S6), where genes can be mapped by more than one SNP set

### Identifying clusters of subjects with distinct character profiles

We identified 342 non-identical but possibly overlapping character sets using the 13 character subscales without knowledge of the genotype. Character sets were labeled by a phenotypic identification “C” to distinguish them from the SNP sets. These fine-grained character sets were nested within five character supersets that were identified by recurrently applying PGMRA to minimize the cophenetic correlation coefficient (Table 2) [62]. In other words, five groups of people had highly distinct character profiles.

**Table 2:** Description of the five character profiles (supersets) and composite character sets identified by PGMRA from profiles of TCI subscales (Y = yes)

Char sets	Supersets	Name	sd1	sd2	sd3	sd4	sd5	co1	co2	co3	co4	co5	st1	st2	st3	Lsd1	Lsd2	Lsd3	Lsd4	Lsd5	Lco1	Lco2	Lco3	Lco4	Lco5	Lst1	Lst2	Lst3	#S	Well Being	Ill Being
C_14_8	1	Resourceful			Y	Y																						Y	79	0.01	0.01
C_10_7	1		Y		Y	Y					Y																		102	0.41	0
C_10_6	1		Y		Y														Y					Y					33	0.03	0.38
C_14_13	2	Organized					Y	Y	Y	Y	Y															Y	Y		92	0.09	0
C_14_9	2						Y	Y	Y		Y	Y	Y			Y											Y		42	0.02	0.29
C_9_8	2			Y	Y	Y	Y	Y	Y	Y	Y			Y											Y	Y		Y	72	0.12	0
C_12_9	2		Y	Y	Y		Y	Y		Y	Y								Y										41	0.54	0
C_6_5	2		Y	Y	Y	Y	Y	Y	Y	Y	Y	Y														Y	Y		56	0.07	0
C_8_7	2		Y	Y	Y	Y	Y	Y	Y	Y	Y	Y														Y	Y	Y	161	0.04	0
C_5_1	2		Y	Y	Y	Y	Y	Y	Y	Y	Y	Y														Y	Y	Y	169	0.05	0
C_3_1	2		Y	Y	Y	Y	Y	Y	Y	Y	Y	Y														Y	Y	Y	293	0	0
C_4_4	2		Y	Y	Y	Y	Y	Y	Y	Y	Y	Y														Y	Y	Y	190	0.01	0
C_7_7	2		Y	Y	Y		Y	Y	Y	Y	Y	Y														Y	Y	Y	101	0.07	0
C_9_6	2		Y	Y	Y		Y	Y	Y	Y	Y	Y							Y							Y	Y	Y	61	0.11	0.02
C_7_5	2					Y		Y	Y	Y																		Y	34	0.5	0.06
C_9_1	2		Y	Y	Y	Y		Y	Y	Y														Y		Y			46	0	0.02
C_15_5	3	Creative									Y				Y														100	0.72	0.03
C_12_7	3												Y		Y														52	0.9	0.04
C_11_3	3												Y	Y	Y														34	0.97	0.03
C_13_1	3				Y									Y	Y														42	0.9	0.02
C_14_1	3		Y	Y								Y	Y		Y				Y		Y						Y		28	0.25	0.11
C_7_2	3		Y	Y	Y		Y				Y	Y	Y	Y	Y														66	0.98	0
C_8_8	3		Y	Y	Y		Y	Y	Y	Y	Y		Y	Y	Y														39	1	0
C_4_3	3		Y	Y	Y		Y	Y	Y	Y		Y	Y	Y	Y														72	0.97	0
C_5_5	3			Y	Y		Y	Y	Y	Y	Y	Y	Y	Y	Y														73	1	0
C_6_1	3			Y	Y	Y		Y	Y	Y	Y	Y	Y	Y	Y														32	1	0
C_3_3	3		Y	Y	Y	Y	Y	Y	Y	Y	Y	Y	Y	Y	Y														135	0.92	0
C_9_2	3												Y	Y	Y	Y			Y					Y					4	0.05	0.2
C_15_7	4	Dependent									Y																	Y	121	0.03	0.02
C_14_5	4					Y												Y										Y	55	0	0.29
C_15_13	4															Y	Y	Y											29	0	0.79
C_12_6	4					Y					Y					Y	Y	Y		Y									44	0.09	0.41
C_4_2	4					Y					Y	Y				Y	Y	Y		Y									40	0	0.45
C_7_4	4										Y	Y					Y	Y				Y				Y	Y		23	0	0.74
C_5_3	4					Y					Y	Y			Y		Y	Y		Y						Y	Y		48	0.02	0.29
C_6_3	4					Y					Y	Y					Y	Y		Y						Y	Y	Y	37	0	0.32
C_9_5	4					Y		Y	Y	Y	Y	Y			Y		Y	Y								Y	Y		31	0	0.26
C_10_2	5	Apathetic																								Y	Y	Y	116	0.01	0
C_11_4	5																									Y	Y	Y	50	0	0.28
C_8_3	5																					Y				Y	Y	Y	39	0	0.64
C_14_11	5																				Y	Y	Y	Y		Y	Y		62	0	0.47
C_10_8	5																Y			Y	Y	Y	Y		Y	Y	Y	Y	33	0	0.79
C_3_2	5															Y	Y	Y	Y	Y	Y	Y	Y	Y	Y				53	0	1
C_12_5	5									Y	Y	Y						Y								Y	Y	Y	22	0	0.09
C_14_7	5														Y	Y	Y	Y	Y										4	0	1
C_13_3	5		Y				Y	Y											Y					Y					4	0	0.25
C_14_3	5																							Y					32	0.06	0.38
C_11_10	5					Y		Y						Y			Y	Y		Y				Y	Y				38	0.03	0.5
C_15_1	5														Y						Y			Y					21	0.14	0.52
C_7_3	5															Y			Y										15	0	1
C_8_6	5															Y		Y	Y										7	0	1
C_9_3	5															Y	Y	Y	Y		Y	Y	Y	Y	Y		Y	Y	7	0	1
C_15_15	5			Y	Y											Y													33	0	0.3
C_11_6	5																		Y					Y					28	0	0.71
C_12_4	5				Y												Y		Y	Y	Y	Y							34	0.03	0.53
		**Consensus sets**	**sd1**	**sd2**	**sd3**	**sd4**	**sd5**	**co1**	**co2**	**co3**	**co4**	**co5**	**st1**	**st2**	**st3**	**Lsd1**	**Lsd2**	**Lsd3**	**Lsd4**	**Lsd5**	**Lco1**	**Lco2**	**Lco3**	**Lco4**	**Lco5**	**Lst1**	**Lst2**	**Lst3**			
		Resourceful	Y		Y	Y																									
		Organized	Y	Y	Y	Y	Y	Y	Y	Y	Y	Y														Y	Y	Y			
		Creative	Y	Y	Y	Y	Y	Y	Y	Y	Y	Y	Y	Y	Y																
		Dependent				Y					Y	Y				Y	Y	Y		Y						Y	Y				
		Apathetic														Y	Y	Y	Y	Y	Y	Y	Y	Y	Y	Y	Y	Y			

TCI subscales are indicated self-directedness (sd1–sd5), cooperativeness (co1–co5), and self-transcendence (st1–st3). Subscale values were divided by median split into high and low scores (distinguished by L before the low scores). The number of subjects in each character set is specified (#S). The probabilities of well-being and ill-being are shown for subjects in each character set (see also Supplementary Table S2)

The people in three of the five character profiles had healthy personalities, which we named resourceful, organized, and creative to be consistent with traditional labels for TCI profiles (Table 2). For example, people with the "organized" character profile were high in most subscales of self-directedness and cooperativeness, but were low in all subscales of self-transcendence (i.e., they were controlling, individualistic, and skeptical). People with the "creative" profile were high in all aspects of character, whereas the "resourceful" were only self-directed.

In addition, there were two profiles of people with unhealthy personalities. The people with a "dependent" character profile were highly forgiving when abused (CO4), conscientiously considerate of others (CO5), self-deprecating (SD4), and otherwise low in self-directedness and self-transcendence. The people with an "apathetic" character were low in all aspects of character development (Table 2).

### Association of SNP sets with character

We tested the association of SNP sets with character. The empirical index of character, a single quantitative measure of character functioning, was more strongly associated with SNP sets than with the average effects of their constituent SNPs according to SKAT (Table 1). Forty-two SNP sets had significant associations with character (*p* < 1E-05). For example, the SNP set G_11_4 has a *p*-value of 1.22 E-19, whereas the best and average SNPs within this set have 9.00 E-05 and 3.29 E-02 *p*-values, respectively (Table 1). SKAT [32] and PLINK [54] methods estimated similar *p*-values for the individual SNPs (R^2^ = 0.99, F statistics, *p* < 3.8 E-46), showing that SKAT did not inflate results.

Forty-two SNP sets significantly associated with character are described in Table 1. We assigned names to the SNP sets based on prominent molecular processes and pathways that distinguished them (Supplementary Table S4). The character-related SNP sets were comprised of networks of SNPs that mapped 727 genes, nearly all of which are known to influence individual differences in brain functions, particularly regulation of neurodevelopment, neuroplasticity, neuroprotection, connectivity, energy metabolism, stress reactivity, resilience, longevity, learning, and memory (Supplementary Tables S5, S6).

### Complex genotypic–phenotypic relationships in personality profiles

We found that 55 of the 342 character sets were significantly associated with particular SNP sets (hypergeometric statistics, 1E-11 < *p* < 1E-03, Table 3). The genotypic–phenotypic relations were complex, demonstrating pleiotropy and heterogeneity. For example, G_5_1 involved neuroplasticity and was frequently associated with dependent character sets, but sometimes with apathetic or creative profiles (Table 3). The 55 character sets were associated with the 42 SNP sets in 128 relationships that were significant by a permutation test (Table 3, empirical *p* < 4.7 E-03).

**Table 3:** The strength of the genotypic–phenotypic relationships among SNP and character sets and their corresponding health measurements

Character sets	Character consensus sets	SNP sets	SNP-set names	Hypergeo-metric C-G	Health measurements of subjects					
					Char sets		SNP sets		Both Sets Jointly	
					Well-being	Ill-being	Well-being	Ill-being	Well-being	Ill-being
C_14_8	Resourceful	G_12_8	Neuroprotection	1.29E-03	0.01	0.01	0.09	0.03	0.09	0.03
C_10_7	Resourceful	G_12_8	Neuroprotection	2.79E-03	0.41	0	0.09	0.03	0.41	0.03
C_10_7	Resourceful	G_33_11	Self-control	3.68E-03	0.41	0	0.26	0.07	0.41	0.07
C_10_6^i^	Resourceful	G_33_19	Res-RNA	4.33E-03	0.03	0.38	0.09	0.12	0.09	0.38
C_14_8	Resourceful	G_11_4	Inositol signaling	4.86E-03	0.01	0.01	0.07	0.04	0.07	0.04
C_4_4	Organized	G_11_4	Inositol signaling	1.26E-11	0.01	0	0.07	0.04	0.07	0.04
C_3_1	Organized	G_11_4	Inositol signaling	7.18E-09	0	0	0.07	0.04	0.07	0.04
C_5_1	Organized	G_11_4	Inositol signaling	2.19E-06	0.05	0	0.07	0.04	0.07	0.04
C_4_4	Organized	G_12_8	Neuroprotection	9.79E-06	0.01	0	0.09	0.03	0.09	0.03
C_3_1	Organized	G_12_8	Neuroprotection	1.38E-05	0	0	0.09	0.03	0.09	0.03
C_8_7	Organized	G_11_4	Inositol signaling	2.78E-05	0.04	0	0.07	0.04	0.07	0.04
C_4_4	Organized	G_10_1	Learning/memory	4.79E-05	0.01	0	0.07	0.06	0.07	0.06
C_3_1	Organized	G_10_1	Learning/memory	9.45E-05	0	0	0.07	0.06	0.07	0.06
C_3_1	Organized	G_21_4	Org1-RNA	1.70E-04	0	0	0.07	0.07	0.07	0.07
C_4_4	Organized	G_8_8	Global inositol/chemokine pathways	1.86E-04	0.01	0	0.08	0.07	0.08	0.07
C_14_13	Organized	G_24_6	Neurogenesis	1.86E-04	0.09	0	0.1	0.08	0.1	0.08
C_4_4	Organized	G_3_1	Inositol calcium signaling	3.38E-04	0.01	0	0.06	0.1	0.06	0.1
C_9_8	Organized	G_12_8	Neuroprotection	4.62E-04	0.12	0	0.09	0.03	0.12	0.03
C_3_1	Organized	G_3_1	Inositol calcium signaling	7.05E-04	0	0	0.06	0.1	0.06	0.1
C_7_7	Organized	G_11_4	Inositol signaling	7.17E-04	0.07	0	0.07	0.04	0.07	0.04
C_5_1	Organized	G_12_8	Neuroprotection	7.58E-04	0.05	0	0.09	0.03	0.09	0.03
C_14_13	Organized	G_12_8	Neuroprotection	8.50E-04	0.09	0	0.09	0.03	0.09	0.03
C_14_13	Organized	G_23_19	Org2-RNA	1.00E-03	0.09	0	0.08	0.03	0.09	0.03
C_5_1	Organized	G_10_1	Learning/memory	1.09E-03	0.05	0	0.07	0.06	0.07	0.06
C_3_1	Organized	G_8_8	Global inositol/chemokine pathways	1.12E-03	0	0	0.08	0.07	0.08	0.07
C_7_7	Organized	G_17_14	Dep-RNA	1.32E-03	0.07	0	0.09	0.15	0.09	0.15
C_15_1i	Organized	G_36_29	Electron transport	1.66E-03	0	0.79	0.08	0.48	0.08	0.79
C_5_1	Organized	G_8_8	Global inositol/chemokine pathways	2.07E-03	0.05	0	0.08	0.07	0.08	0.07
C_14_13	Organized	G_11_4	Inositol signaling	2.33E-03	0.09	0	0.07	0.04	0.09	0.04
C_9_1i	Organized	G_38_10	Org5-RNA	2.34E-03	0	0.02	0.15	0.19	0.15	0.19
C_7_5i	Organized	G_35_12	Org4-RNA	2.43E-03	0.5	0.06	0.16	0.09	0.5	0.09
C_7_7	Organized	G_12_8	Neuroprotection	2.50E-03	0.07	0	0.09	0.03	0.09	0.03
C_5_1	Organized	G_17_14	Dep-RNA	3.00E-03	0.05	0	0.09	0.15	0.09	0.15
C_6_5	Organized	G_11_4	Inositol signaling	3.04E-03	0.07	0	0.07	0.04	0.07	0.04
C_12_9	Organized	G_7_3	Neurogenesis	3.13E-03	0.54	0	0.17	0.36	0.54	0.36
C_8_7	Organized	G_10_1	Learning/memory	3.54E-03	0.04	0	0.07	0.06	0.07	0.06
C_4_4	Organized	G_24_6	Neurogenesis	3.63E-03	0.01	0	0.1	0.08	0.1	0.08
C_14_9	Organized	G_31_8	Neurotrophin	3.65E-03	0.02	0.29	0.09	0.54	0.09	0.54
C_14_9	Organized	G_7_3	Neurogenesis	3.66E-03	0.02	0.29	0.17	0.36	0.17	0.36
C_9_6	Organized	G_27_25	Org3-RNA	3.84E-03	0.11	0.02	0.11	0.07	0.11	0.07
C_8_7	Organized	G_8_8	Global inositol/chemokine pathways	3.99E-03	0.04	0	0.08	0.07	0.08	0.07
C_12_9	Organized	G_9_8	Neuroregulation	4.96E-03	0.54	0	0.17	0.12	0.54	0.12
C_3_3	Creative	G_7_3	Neurogenesis	1.22E-06	0.92	0	0.17	0.36	0.92	0.36
C_3_3	Creative	G_5_1	CDK neuroplasticity	2.00E-06	0.92	0	0.17	0.4	0.92	0.4
C_15_5	Creative	G_3_2	PAK-neuroprotection	6.63E-06	0.72	0.03	0.18	0.24	0.72	0.24
C_4_3	Creative	G_20_2	Enhanced memory	1.13E-05	0.97	0	0.24	0.12	0.97	0.12
C_14_1	Creative	G_7_3	Neurogenesis	2.80E-05	0.25	0.11	0.17	0.36	0.25	0.36
C_11_3	Creative	G_33_15	ERK-neurodevelopment	3.99E-05	0.97	0.03	0.27	0.23	0.97	0.23
C_8_8	Creative	G_20_2	Enhanced memory	6.49E-05	1	0	0.24	0.12	1	0.12
C_3_3	Creative	G_20_2	Enhanced memory	9.26E-05	0.92	0	0.24	0.12	0.92	0.12
C_5_5	Creative	G_20_2	Enhanced memory	1.41E-04	1	0	0.24	0.12	1	0.12
C_4_3	Creative	G_33_15	ERK-neurodevelopment	1.65E-04	0.97	0	0.27	0.23	0.97	0.23
C_5_5	Creative	G_33_15	ERK-neurodevelopment	1.78E-04	1	0	0.27	0.23	1	0.23
C_12_7	Creative	G_33_15	ERK-neurodevelopment	3.21E-04	0.9	0.04	0.27	0.23	0.9	0.23
C_4_3	Creative	G_7_3	Neurogenesis	3.65E-04	0.97	0	0.17	0.36	0.97	0.36
C_5_5	Creative	G_7_3	Neurogenesis	4.21E-04	1	0	0.17	0.36	1	0.36
C_6_1	Creative	G_33_15	ERK-neurodevelopment	5.02E-04	1	0	0.27	0.23	1	0.23
C_5_5	Creative	G_19_5	DARPP320-Neuroplasticity	5.30E-04	1	0	0.16	0.22	1	0.22
C_3_3	Creative	G_19_5	DARPP320-neuroplasticity	7.66E-04	0.92	0	0.16	0.22	0.92	0.22
C_3_3	Creative	G_33_15	ERK-neurodevelopment	8.46E-04	0.92	0	0.27	0.23	0.92	0.23
C_4_3	Creative	G_12_1	Episodic learning	9.12E-04	0.97	0	0.2	0.06	0.97	0.06
C_7_2	Creative	G_33_15	ERK-Neurodevelopment	9.82E-04	0.98	0	0.27	0.23	0.98	0.23
C_7_2	Creative	G_36_27	Aging regulation	1.18E-03	0.98	0	0.33	0.22	0.98	0.22
C_13_1	Creative	G_20_2	Enhanced memory	1.23E-03	0.9	0.02	0.24	0.12	0.9	0.12
C_13_1	Creative	G_33_15	ERK-neurodevelopment	1.44E-03	0.9	0.02	0.27	0.23	0.9	0.23
C_3_3	Creative	G_12_1	Episodic learning	1.59E-03	0.92	0	0.2	0.06	0.92	0.06
C_12_7	Creative	G_12_1	Episodic learning	2.21E-03	0.9	0.04	0.2	0.06	0.9	0.06
C_3_3	Creative	G_9_8	Neuroregulation	2.27E-03	0.92	0	0.17	0.12	0.92	0.12
C_3_3	Creative	G_28_11	Sensory transduction	3.14E-03	0.92	0	0.22	0.06	0.92	0.06
C_13_1	Creative	G_28_11	Sensory transduction	3.17E-03	0.9	0.02	0.22	0.06	0.9	0.06
C_4_3	Creative	G_9_8	Neuroregulation	3.32E-03	0.97	0	0.17	0.12	0.97	0.12
C_5_5	Creative	G_12_1	Episodic learning	3.32E-03	1	0	0.2	0.06	1	0.06
C_9_2i	Creative	G_34_13	CREB-Episodic learning	4.05E-03	0.05	0.2	0.05	0.29	0.05	0.29
C_12_7	Creative	G_7_3	Neurogenesis	4.10E-03	0.9	0.04	0.17	0.36	0.9	0.36
C_12_7	Creative	G_19_5	DARPP320-Neuroplasticity	4.22E-03	0.9	0.04	0.16	0.22	0.9	0.22
C_11_3	Creative	G_9_8	Neuroregulation	4.24E-03	0.97	0.03	0.17	0.12	0.97	0.12
C_15_7	Dependent	G_11_4	Inositol signaling	1.80E-06	0.03	0.02	0.07	0.04	0.07	0.04
C_12_6	Dependent	G_31_8	Neurotrophin	8.93E-06	0.09	0.41	0.09	0.54	0.09	0.54
C_12_6	Dependent	G_41_33	GPCR neuroplasticity	1.18E-05	0.09	0.41	0.11	0.21	0.11	0.41
C_15_13	Dependent	G_31_8	Neurotrophin	6.14E-05	0	0.79	0.09	0.54	0.09	0.79
C_4_2	Dependent	G_28_15	Histone methylation	6.90E-05	0	0.45	0.08	0.38	0.08	0.45
C_9_5	Dependent	G_7_2	GPCR dysregulation	1.01E-04	0	0.26	0.09	0.23	0.09	0.26
C_15_7	Dependent	G_8_8	Global inositol/chemokine pathways	2.03E-04	0.03	0.02	0.08	0.07	0.08	0.07
C_15_13	Dependent	G_28_15	Histone methylation	2.92E-04	0	0.79	0.08	0.38	0.08	0.79
C_4_2	Dependent	G_5_1	CDK neuroplasticity	3.98E-04	0	0.45	0.17	0.4	0.17	0.45
C_14_5	Dependent	G_7_7	Olfaction	8.92E-04	0	0.29	0.03	0.1	0.03	0.29
C_15_7	Dependent	G_17_14	Dep-RNA	1.01E-03	0.03	0.02	0.09	0.15	0.09	0.15
C_6_3	Dependent	G_17_14	Dep-RNA	1.09E-03	0	0.32	0.09	0.15	0.09	0.32
C_12_6	Dependent	G_7_3	Neurogenesis	1.21E-03	0.09	0.41	0.17	0.36	0.17	0.41
C_5_3	Dependent	G_5_1	CDK neuroplasticity	1.43E-03	0.02	0.29	0.17	0.4	0.17	0.4
C_15_13	Dependent	G_5_1	CDK neuroplasticity	1.83E-03	0	0.79	0.17	0.4	0.17	0.79
C_5_3	Dependent	G_7_3	Neurogenesis	2.32E-03	0.02	0.29	0.17	0.36	0.17	0.36
C_9_5	Dependent	G_5_1	CDK neuroplasticity	2.63E-03	0	0.26	0.17	0.4	0.17	0.4
C_4_2	Dependent	G_7_3	Neurogenesis	2.66E-03	0	0.45	0.17	0.36	0.17	0.45
C_6_3	Dependent	G_7_7	Olfaction	2.73E-03	0	0.32	0.03	0.1	0.03	0.32
C_4_2	Dependent	G_31_8	Neurotrophin	2.93E-03	0	0.45	0.09	0.54	0.09	0.54
C_6_3	Dependent	G_22_6	Blood-brain barrier	3.40E-03	0	0.32	0.08	0.16	0.08	0.32
C_4_2	Dependent	G_41_33	GPCR neuroplasticity	3.44E-03	0	0.45	0.11	0.21	0.11	0.45
C_7_4	Dependent	G_5_1	CDK neuroplasticity	3.56E-03	0	0.74	0.17	0.4	0.17	0.74
C_14_5	Dependent	G_28_15	Histone methylation	3.73E-03	0	0.29	0.08	0.38	0.08	0.38
C_3_2	Apathetic	G_5_1	CDK neuroplasticity	6.13E-10	0	1	0.17	0.4	0.17	1
C_3_2	Apathetic	G_7_3	Neurogenesis	4.61E-08	0	1	0.17	0.36	0.17	1
C_3_2	Apathetic	G_28_15	Histone methylation	2.32E-05	0	1	0.08	0.38	0.08	1
C_9_3i	Apathetic	G_26_14	Glucose transport	3.12E-04	0	1	0.09	0.24	0.09	1
C_3_2	Apathetic	G_7_2	GPCR dysregulation	4.18E-04	0	1	0.09	0.23	0.09	1
C_10_2	Apathetic	G_11_4	Inositol signaling	4.25E-04	0.01	0	0.07	0.04	0.07	0.04
C_12_4i	Apathetic	G_35_4	O-linked glycosylation	4.30E-04	0.03	0.53	0.05	0.24	0.05	0.53
C_11_4	Apathetic	G_16_9	Olfactory signaling	5.78E-04	0	0.28	0.08	0.17	0.08	0.28
C_7_3i	Apathetic	G_36_29	Electron transport	5.96E-04	0	1	0.08	0.48	0.08	1
C_10_8	Apathetic	G_5_1	CDK neuroplasticity	6.43E-04	0	0.79	0.17	0.4	0.17	0.79
C_14_7i	Apathetic	G_36_29	Electron transport	7.85E-04	0	1	0.08	0.48	0.08	1
C_11_10i	Apathetic	G_36_29	Electron transport	8.39E-04	0.03	0.5	0.08	0.48	0.08	0.5
C_11_6i	Apathetic	G_20_3	Fatty acid oxidation	1.06E-03	0	0.71	0.03	0.33	0.03	0.71
C_10_8	Apathetic	G_31_8	Neurotrophin	1.21E-03	0	0.79	0.09	0.54	0.09	0.79
C_15_15i	Apathetic	G_41_40	Apha-RNA	1.61E-03	0	0.3	0.09	0.03	0.09	0.3
C_14_11	Apathetic	G_28_15	Histone methylation	2.13E-03	0	0.47	0.08	0.38	0.08	0.47
C_3_2	Apathetic	G_40_26	Dopamine-feedback	2.41E-03	0	1	0.08	0.36	0.08	1
C_10_2	Apathetic	G_9_3	Pyrimidine metabolism	2.60E-03	0.01	0	0.12	0.04	0.12	0.04
C_13_3i	Apathetic	G_26_14	Glucose transport	2.67E-03	0	0.25	0.09	0.24	0.09	0.25
C_8_6i	Apathetic	G_36_29	Electron transport	2.69E-03	0	1	0.08	0.48	0.08	1
C_10_8	Apathetic	G_19_9	Aurora-B	3.25E-03	0	0.79	0.1	0.4	0.1	0.79
C_10_8	Apathetic	G_7_3	Neurogenesis	3.47E-03	0	0.79	0.17	0.36	0.17	0.79
C_10_8	Apathetic	G_23_2	Biogenic synthesis	3.56E-03	0	0.79	0.05	0.29	0.05	0.79
C_11_6i	Apathetic	G_36_29	Electron transport	3.88E-03	0	0.71	0.08	0.48	0.08	0.71
C_12_5	Apathetic	G_7_2	GPCR dysregulation	3.99E-03	0	0.09	0.09	0.23	0.09	0.23
C_10_2	Apathetic	G_10_1	Learning/memory	4.03E-03	0.01	0	0.07	0.06	0.07	0.06
C_8_3	Apathetic	G_21_3	Cellular Senescence	4.17E-03	0	0.64	0.1	0.23	0.1	0.64
C_14_3i	Apathetic	G_26_14	Glucose transport	4.43E-03	0.06	0.38	0.09	0.24	0.09	0.38

Association is measured by Fisher's exact test (hypergeometric). Probabilities of well-being and ill-being are given for subjects in the character sets, the SNP sets, and subjects identified in both jointly. ^i^Indicates character sets that are more specific than their parental sets, which are also selected

SNP sets (Fig. 1b, Supplementary Figure 2A) often had similar character profiles associated with particular molecular processes (Table 3, Supplementary Tables S4, S7). For example, the organized profile was strongly associated with many SNP sets involving the regulation of inositol–calcium signaling for obtaining food and other goals (e.g., G_8_8, G_11_4) and for neuroprotection against injury (G_12_8). SNP sets regulating episodic learning and hippocampal neurogenesis (e.g., G_7_3, G_12_1) were associated with a creative profile.

### Relations among SNP sets to one another and to molecular processes

We found 12 single and disjoint nodes, and at least three subnetworks composed of highly connected nodes, shown in Fig. 1b and Supplementary Figure S3A. These networks were relatively disjoint (i.e., sharing few SNPs and subjects; see Supplementary Information 9. Identification of Sub-networks), suggesting that these are distinct antecedents of personality. These nearly disjoint networks vary in size and complexity: one subnetwork connected eight SNP sets (Supplementary Figure S3A), whereas others had only a single SNP set.

One network contained SNP sets primarily connected by shared SNPs, but not subjects (e.g., G_10_1 learning/memory and G_7_7 olfaction, Fig. 1b), as expected when the same SNPs had different allele values. This network was associated with dependent and organized personality profiles (Fig. 1b).

Both shared subjects and SNPs connected the other two networks (Fig. 1b), as occurs when one network is a subset of another. The first network was primarily composed of organized (e.g., components of inositol signaling by G_11_4, G_8_8, G_3_1) and apathetic (e.g., G_21_3 cellular senescence, G_7_2 GPCR dysregulation) profiles. The second network displayed creative (e.g., G_3_2, G_7_3, G_9_8) and dependent (e.g., G_38_8, G_5_1) profiles.

Finally, some SNP sets within a network do not share SNPs, but independently specify almost the same individuals (e.g., G_8_8 inositol/chemokine signaling, G_7_2 GPCR dysregulation, Fig. 1b), as expected when distinct subsets of genotypic features influence a common pathway or consequence.

### Heterogenic pathways influence the same character trait

The genes associated with each of the five character profiles are largely different. In all, 68% of the 727 genes associated with character were unique to a single character profile: 208 with organized, 89 with creative, 70 with dependent, and 130 with apathetic (Supplementary Table S8). Consequently, there were multiple groups of genes that lead to each individual character trait, as depicted in Fig. 1f. For example, high self-directedness occurs in individuals with the resourceful, organized, and creative profiles, even though these profiles have different genetic backgrounds. Put another way, individual character traits were genetically more heterogeneous than the multidimensional character profiles.

We refer to the multiple genotypic–phenotypic networks that contribute to individual traits as a pipeline, as outlined in Fig. 1f. Detailed descriptions of the specific genes and molecular processes we found in the pipelines for each of the three character traits are presented in Supplementary Tables S9–S12.

### Complex genotypic–phenotypic relationships influence health status

The combination of genotypic and phenotypic information provided more information than either alone for both well-being (Fig. 1g vs. Fig. 1h) and ill-being (Supplementary Figures S4 vs. S5). When health status was based on the joint relationship of SNP sets and character sets, all five character profiles were well distinguished in terms of the probabilities of ill-being (*p* < 3.89E-26, ANOVA statistics, Fig. 1c) and well-being (*p* < 3.68E-65, ANOVA, Fig. 1d). In contrast, when health status was based on character scores only, the probability of ill-being was greater in only two profiles and that of well-being was greater in only one profile (Supplementary Figure S6).

We identified candidate regulatory genes that we called switch genes because of their relationship to changes in health status among people with the same character profile (Fig. 1e). For example, all apathetic SNP sets were associated with ill-being except G_9_3, which was associated with well-being. In contrast, the creative SNP sets were associated with well-being except for G_7_7, which was associated with ill-being. The 150 switch genes included 50% protein coding genes, 18% RNA genes, 15% pseudogenes, 3% transcription factors, and 4% others (Supplementary Table S13).

Overall about 67% of the 727 genes associated with character sets may be involved in regulatory processes: these included transcriptional regulators (10%), lncRNAs (24%), other RNA genes (6%), and targets of microRNAs (27%), as identified in the TRANSFAC^®^ release 2017.1 database (Supplementary Table S14). We identified two microRNAs (MIR431, MIR1762) in association with character, and they target 74 and 119 of the 727 genes we found associated with character in TRANSFAC, respectively. In particular, lnc RNAs were more commonly associated with character only then with temperament and character, whereas protein-coding genes were more commonly associated with both temperament and character, as shown in Fig. 2a, b.

### Replication of results in two independent samples

We tested the replicability of our findings in the Finnish study by carrying out the same analyses in the German and Korean samples. In all, 95% of the 42 SNP sets associated with character sets in the Finnish sample were identified in one or both of the replication samples: 36 were identified in both the Korean and German samples, three in the Korean sample only, and one in the German sample only (Supplementary Table S15). In addition, 96% of the 55 character sets associated with SNP sets in the Finnish sample were replicated in one or both of the replication samples: 46 in both, six in Korean sample only, and one in the German sample only (Table S16). The genotypic–phenotypic relations between SNP and character sets identified in the Finnish sample closely matched those observed in the Korean study (94%) and in the German (84%) study (Table S17). The replication of the 25 character sets associated with ill-being in the Finnish sample was reduced in the German sample (72%) compared with the Korean sample (84%)(ANOVA, *p* = 0.01), as expected because the Germans had been screened to exclude psychopathology, including personality disorders, in themselves or their first-degree relatives (Supplementary Figure S7). The strength of the identity of replicated sets was calculated using hypergeometric statistics and multi-objective optimization techniques (see Pareto values in Supplementary Tables S18, S19).

We also surveyed prior literature reporting associations with TCI character-related keywords systematically from PubMed, and identified genes that had been reported to be associated with one or more of the TCI character traits in one or more investigations (Supplementary Tables S20, S21). We found that 116 of our detected genes were related to genes, family of proteins, or pathways of genes previously associated with TCI traits (Supplementary Table S20). Among the genes in character-related SNP sets, we also detected 74% of the 111 genes that had been previously associated with TCI traits, and 75% of the 63 genes that had previously been reported in association with TCI character traits (Supplementary Table S21). Considering all genes previously related to the TCI (Supplementary Table S21), we recovered seven genes with the same exact name, another 34 variants from the same family of proteins, and another 41 genes in the same KEGG pathway previously reported.

### Estimation of heritability and environmental influences

The heritability of character controlling for outliers was estimated as 57% in the Finns, 58% in the Germans, and 50% in the Koreans (Supplementary Table S22). In addition, 95% of the SNP sets were strongly associated with the empirical character index (5E-11 > *p*-value > 5E-77). In other words, the SNPs that comprise different SNP sets strongly distinguished the character values of the subjects in each set, indicating that each individual SNP set contributed significantly to explain the total distributed heritability (Supplementary Section 9). Consequently, when the genotypic sets were used to classify the well-being and ill-being of the subjects as measured by their character values, the predicted values were highly accurate (average areas under curve of the classifications were 0.928 and 0.932, respectively) (Supplementary Figure S9).

We also considered environmental influences in the Finnish sample. There were direct associations of sets of environmental influences in childhood and adulthood with character sets (Supplementary Table S23A) and with SNP sets (Supplementary Table S23B). The impact of these correlations was small, so the heritability estimate was still 56% in the Finnish sample when adjusted for gene-environment correlation (Supplementary Table S23C). In addition, five novel associations between SNP sets and character sets emerged when environmental influences were used as mediators: years of education in childhood and stressful life events in adulthood had significant effects on organized and dependent character profiles (Supplementary Table S23D, *p* < 2.9 to 8.4 E-03).

## Discussion

This is the first data-driven study to examine the genotypic–phenotypic architecture of human character traits, which are the self-regulatory components of personality that modulate physical, mental, and social well-being [48, 65]. As such, it represents a pioneering effort to describe the psychobiology of character as a complex network of genotypes with specific molecular processes and neuronal functions that regulate personality development. We explained 50–58% of the heritability of human character and replicated our results in independent samples, thereby accounting for nearly all the heritability expected from twin studies.

### Complexity of genotypic–phenotypic pipelines

We observed that 68% of the 727 genes for character were unique to a single character profile and were regulated by distinct molecular processes and neuronal functions. Such minimal overlap in genes and molecular mechanisms between personality profiles is very surprising from a trait perspective. For example, both the organized and creative character profiles are high in self-directedness and cooperativeness, and differ only in self-transcendence. The resourceful profile differs from the apathetic profile only in being high in self-directedness. Thus, we hypothesize that people can become highly self-directed by multiple mechanisms: a creative or intuitive route involving enhancing self-awareness in episodic memory, an organized or analytical route involving executive control of what is known from past experience, and/or taking initiative by learned resourcefulness.

Likewise, there are three or more routes via distinct genetic pipelines to cooperativeness and/or self-transcendence. Consequently, individual personality traits are genetically heterogeneous and their development depends on multiple mechanisms that can only be distinguished by consideration of the whole person. Individual traits may still be important for study of development or treatment, but they do not appear to be the fundamental building blocks of personality.

### Regulatory processes and functions associated with character

We observed that 67% of the 727 character genes were involved in regulatory systems. In particular, lncRNAs were more common in association with character only than with both temperament and character (Fig. 2a, b). The identified genes are reported to influence neuroplasticity, energy metabolism, and the regulation of adaptations to a wide variety of biological, psychological, and social stressors through processes for intentional goal-seeking, self-control, empathy, and episodic memory (Table 1). These genetic findings are supported by independent neuroimaging findings that TCI character traits are associated with brain networks for these same intentional and meta-cognitive functions [24–27].

An interesting sign of the high predictability of variability in health status was our finding that a few genes could dramatically alter the health status of people with each specific SNP set, including 150 putative switch genes across all 42 SNP sets. The dramatic effect that a few switch genes can have on overall health status is further evidence of the importance of epistasis for understanding personality and its development.

### Strengths and limitations

Our unbiased analytical PGMRA method used deep cluster analysis to identify association between possibly interactive sets of features instead of between individual SNPs or character traits. The results were strongly replicated in independent samples, demonstrating remarkable robustness. Furthermore, the neuronal functions of the identified genes are supported by independent research about brain networks related to TCI character.

Our initial pool of SNPs was preselected to be the best candidates to have additive and/or non-additive effects on character. The threshold for possible association (*p*-value of 0.01 without Bonferroni correction) in our initial pool of SNPs was more than six orders of magnitude below what is required for genome-wide significance. We sought to evaluate the cooperative effects of groups of SNPs with possible non-additive gene–gene interactions and those with strong additive effects individually (i.e., very low *p*-values). Therefore, we included SNPs that were either weakly or strongly associated with character singly, and then compared their significance as a group vs. that of the best SNP within the group. Consequently, these candidate SNPs may have no main (additive) effect on the phenotype at all, but when organized as SNP sets, they presented consistent evidence of epistasis (i.e., each SNP set had stronger associations with character than their best single constituents). In addition, the SNPs we identified were sufficient to account for nearly all the heritability expected from twin studies (about 50%), which includes both additive and non-additive effects.

Our findings are based on associations only, which precludes definite conclusions about causation. Nevertheless, the circumstantial evidence for our causal hypotheses is strong and merits further testing.

### Conclusions and recommendations for future research

We were able to characterize and replicate the complexity of the genotypic–phenotypic risk architecture of self-regulatory character traits in three large samples. Our findings demonstrate that data-driven analysis of the architecture of genotypic–phenotypic relationships enables investigators to overcome the hidden heritability problem (i.e., the consistent inability to account for most of the heritability of complex traits when only the average effects of genes are considered). We conclude that self-regulatory personality traits are strongly influenced by organized interactions among more than 700 genes, despite variable cultures and environments. We recommend studies that dissect detailed phenomic and genomic data, including brain images and physiological measurements, and integrate these in a multi-faceted view of each person. We also recommend an extended replicability analysis, in which a marker can be replicated at different multi-omic levels, such as genes, family of proteins, or pathways. The precision of our person-centered approach now allows such in-depth analysis and replication, even for complex traits in moderate-sized samples.

## Electronic supplementary material

Supplementary Information

Supplementary Information (Marked)

Figure S1A

Figure S1B-C

Figure S1D-F

Figure S2

Figure S3

Figure S4

Figure S5

Figure S6

Figure S7

Table S1

Table S2

Table S3

Table S4

Table S5

Table S6

Table S7

Table S8

Table S9

Table S11

Table S10

Table S12

Table S13

Table S14

Table S15

Table S16

Table S17

Table S18

Table S19

Table S20

Table S21

Table S22

Table S23
