# Supplementary material for: Uncovering the complex genetics of human character
Source: Mol Psychiatry. 2018 Oct 3;25(10):2295–312. doi: 10.1038/s41380-018-0263-6 (PMC7515844; doi:10.1038/s41380-018-0263-6)
Supplement: Supplementary file 1 — Supplementary Information [file 41380_2018_263_MOESM1_ESM.docx]

**Supplementary Information**

1. Description of the TCI scales and subscales

2. Comparability of TCI assessment in 3 samples

3. Pre-selection of SNPS

(i) The analysis of the covariate effect

(ii) Pre-discovery analysis of covariate effect performed by Plink

(iii) Post-discovery analysis of covariate effect performed by SKAT

4. Advantages of SNP-set analysis over alternative tests of single and multiple markers

5. The PGMRA method: Summary

6. The PGMRA algorithm

(i) Identify optimal genotypic and/or phenotypic sets (Implemented in the PGMRA web server ^1^).

*Mathematical description of the NMF*

*Transforming NMF k factors into sub-matrices or biclusters which are interpreted*

*as SNP or phenotypic sets.*

*Decomposing the data into a multilevel family of sub-matrices*

*Learning the W and H matrices of FNMF*

*Optimally assembling the families of sub-matrices*.

(ii) Perform a statistical analysis of SNP sets (accessed via the PGMRA web server ^1^).

(iii) Calculate the frequency of subject status within a SNP set

(iv) Discover and encode relations among SNP sets into topologically organized networks

(v) Identify optimal and significant phenotypic sets (Implemented in the PGMRA web server ^1^).

(vi) Identify optimal genotype-phenotype latent architectures

(vii) Semi-supervised learning: transforming unsupervised knowledge bases into supervised classifiers

*PGMRA semi-supervised classifier*.

*kNN classifier*.

*k-Fold Cross Validation*.

*Metrics.*

*Imbalanced datasets*.

(viii) Bioinformatics analysis of the SNP set-related genes, their molecular consequences and pathways

7. Estimation of heritability

8. Replicability of results

(i) Statistical replicability of sets of markers

(ii) Justification of the statistical replicability of sets of markers

(iii) Hierarchical and multi-omic replicability of markers

9. Analysis of results

(i) QQ Plots and covariate analysis

(ii) Statistical analysis: evaluation of SNP and phenotypic sets by additional tests

(iii) Semi-Supervised approach: Classifying subject phenotype based on the genotype

**1. Description of the TCI scales and subscales**

Descriptors of high and low scorers on each of the subscales of the 7 TCI higher-order dimensions are presented in Table S1. All subscales were quantified as the average score per item in that subscale (so that 1 indicated an extreme low score, 3 a score near average, and 5 an extreme high score). For purposes of pattern recognition of profiles, medians for each subscale were calculated and those above the median were rated high and those below were rated low.

**2. Comparability of TCI assessment in three samples**

The Finnish longitudinal study involved 2,149 subjects (assessed in 1997, 2001, 2007, 2012), who completed the original TCI with 240 items using a 5-point Likert scale instead of the original true-false answer format ^2, 3^. The averages of the scales and subscales scores across the four assessment occasions were utilized. The German subjects answered the same 240 TCI items using the original true-false format. Strong concordance between alternative formats in German and other languages has been previously demonstrated ^4^. The Korean subjects used a short form made up of the 140 items with the strongest correlation with the full scales and using the same 5-point Likert format used in Finland ^5, 6^.

**3. Pre-selection of SNPS**

Our samples were treated as representative of the general population for initial analysis because when recruited they were not subdivided experimentally into unhealthy cases and healthy controls. To select an initial subset of SNPs and discard non-relevant SNP observations, we first cleaned the SNP data in the sample as described in ^7^. Then, we selected SNPs with a high threshold (p-value<0.01 without Bonferroni correction) from a logistic or continuous regression calculated by Plink against the empirical character phenotypic index described below (see below item (a) and Calculation of cluster rankings). The quality control (QC) of the genotypic data was performed following the steps detailed in references, removing consequently all the SNPs satisfying the following conditions: SNP call rate < 95% in either datasets, Hardy-Weinberg (HWE) p‐value < 10E‐6, Minor Allele Frequency (MAF) < 1%, and >1 discordant genotypes in either sample duplicates. To select the subject status for the regression, and because we did not have cases and controls, we calculated 3 indicators of variability in personality that could be estimated consistently in all three samples we analyzed (see below). Then, a subset of SNPs were pre-selected to reduce the large search space using the Plink software suite ([3](#_ENREF_3)), taking sex and ancestry as covariates (see below), and using a generously inclusive threshold (p-value < 0.01 without Bonferroni correction) for association with character.

(a) Derivation of the empirical character index: First, we calculated a purely empirical (i.e., agnostic and data-driven) indicator of character functioning. We clustered subjects corresponding to the 13 character subscales and assigned each subject the number of the cluster to which they belonged (as described in the next paragraph). The result was a single empirical index of character cluster membership that served as a comprehensive measure of variability in character.

To calculate the cluster rankings we applied hierarchical agglomerative clustering (Statistical Toolbox, Matlab 2007b) with a complete linkage method and correlation similarity measurement to group SNP, phenotypic, or environmental sets by their shared subjects using hypergeometric statistics. The function that controls the vertical order in which a row is plotted (Spotfire Decision Site 9.1.2) in a hierarchical clustering is defined as follows.

Given two sub-clusters within a cluster (there are always exactly two sub-clusters considered at each step), both sub-clusters are weighted and the sub-cluster with the highest weight is placed above the other sub-cluster. This function is systematically applied until a single cluster containing all rows is obtained. To calculate the weight w_3_ of a new cluster C_3_ formed from two sub-clusters C_1_ and C_2_ with a weight of w_1_ and w_2_, and each containing n_1_ and n_2_ rows, the following expression is used:
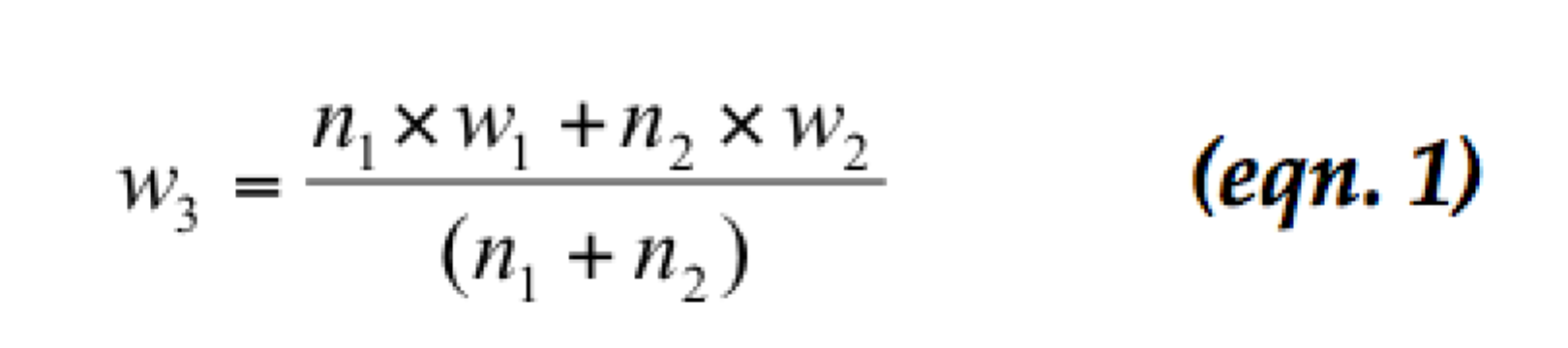


The weight of a sub-cluster with a single row is calculated as the average value of its columns.

(b) Derivation of Dichotomous Indices of Well-being and Ill-being: Second, we calculated a partly theoretically-based indicator based on extensive prior work showing that high values on product of each of the three TCI character scales (that is, SDxCOxST) was associated empirically with positive health whereas low scores on the sum of Self-directedness (SD) and Cooperativeness (CO) was associated empirically with ill health. Specifically, we calculated well- and ill-being indices (see below second item) where subjects in the first and bottom deciles of SDxCOxST and SD+CO, respectively (see Table S2) were labeled as class ‘1’ whereas the remaining subjects were labeled as class ‘0’. Thus being extremely high or low was distinguished from being intermediate for pre-selection purposes.

(c) Derivation of Semi-quantitative Indices of Well-being and Ill-being: Third, we used the partly theoretical indicators to identify intermediate variation along with extreme variability in a semi-quantitative manner. As an extension of (b), we distinguished six equal-sized classes instead of a dichotomous classification (see Table S2, e.g. 0-16% labeled as 1, 16-32% as 2, …). Extreme values 1 and 6 from analysis (c) contained most of the same subjects as were detected in the dichotomous (c) classification.

The empirical index calculated in (a) characterizes all the subjects, not only the extreme subjects. Moreover, this index is a single quantitative index, whereas the well and ill-being indices mixed individuals with either extreme high or extreme low well-being. Finally, the index in (a) can be re-calculated for Character or Environmental variables and used as a measurement inherent to the sample, whereas the well- and ill-being indices are all based on Character subscales because prior research had established character is the self-regulatory component of personality, which largely accounts for differences between people with healthy (mature) and unhealthy (immature) adaptation.

(d) Application of the Indices: We used the empirical character index (i.e., procedure (a)) in the classification of SNP sets by SKAT. Note that SKAT for single SNPs provides a regression similar to that of Plink for individual SNPs ^7^. Then, and independently, we tested the correlations between results when well- and ill-being indices were utilized in Plink and SKAT. The minimum p-value of all indices from (b) and (c) had a very strong correlation with the index from (a) (p value < E-20, RMSE 0.03). Thus our empirical character index and the well- and ill-being measurements are highly correlated indicators of personality variability, so that the empirical indicator was able to identify about 95% of the pre-selected SNPs associated with character.

**(i) The analysis of the covariate effect**

We accounted for ethnicity by using first three principal components (PCs) that account for ancestral stratification of SNP genotypes and sex ^8, 9^. We took stratification into account both before and after the machine learning process. First, we accounted for covariates when selecting the initial set of individual input SNPs by using Plink ^10^. Second, after selecting the constituents of the SNP sets, each SNP set was evaluated by SKAT ^11, 12^, which accounts for covariates when sets of SNPs are assessed.

**(ii) Pre-discovery analysis of covariate effect performed by Plink**

Population stratification requires a covariate study before performing the statistical analyses. The magnitude of the effects of the population stratification depends on sample size, heritability, linkage disequilibrium structure and the number of causal variants ^6^. Confounding bias occurs when a risk factor is also associated with the marker, as is observed when both disease and allelic frequency are correlated through ethnicity. This problem appears more markedly in the context of GWAS since these studies require a very large sample and are often carried out in different countries. Population stratification results in the inflation of p-values. This inflation can be detected and corrected for when testing for alleles that are associated with disease. The accepted way to correct for this effect is the use of covariates in the statistical analysis. It is important to limit the number of covariates because too many covariates can decrease the power of true detection ^13^.

In this study we performed an analysis of covariates based on Quantile-Quantile Plots (QQ Plots) and Lambda genomic control ($\text{λ}$) values to ensure the quality of adjustment. The QQ Plot is a graphical technique for determining if two data sets come from populations with a common distribution. If two sets come from a population with the same distribution, the points should fall approximately along a 45-degree reference line that is also plotted ^14^. The $\text{λ}$ is used to calculate the genomic inflation factor. The expected $\text{λ}$ value is 1. If the value is greater than 1, then this may be evidence for some systematic bias that needs to be corrected in the study.

**(iii) Post-discovery analysis of covariate effect performed by SKAT**

SKAT is a SNP-set level test for association between a set of rare (or common) variants and dichotomous or quantitative phenotypes. SKAT aggregates individual score test statistics of SNPs in a SNP set and efficiently computes SNP-set level p-values while adjusting for covariates, such as PCs to account for population stratification. For post-discovery analysis, we used 3 PCs previously described and gender as covariates. SKAT also properly accounts for multiple comparisons ^1, 11, 12^.

**4. Advantages of SNP-set analysis over alternative tests of single and multiple markers**

In order to understand the novel design and methods of analysis of our study, it is useful to place it in the context of its strong advantages over the fundamental limitations of earlier methods in terms of their reproducibility, interpretability, and power. A long record of peer-reviewed work documents the advantages of SNP-set analysis in GWAS over alternative analyses of individual and multiple SNPs, particularly in adequately handling complex phenotypic-genotypic relationships influenced by epistasis and genetic variants in linkage disequilibrium (LD).

Analyses of associations with individual SNPs are often limited by poor reproducibility; that is, many of the highly ranked SNPs in the discovery phase are false positives and cannot be replicated or otherwise validated. This is largely due to the restricted power to detect SNPs with small effects that are truly associated with the outcome. Unfortunately, the individual SNPs that are genotyped on uncustomized GWAS platforms often show only modest effects with any particular phenotype. One explanation for this is that the true causal SNP is rarely genotyped but there are typed SNPs that are in linkage disequilibrium (LD) with the causal SNP. In this case, when individual-SNP analysis is used, the typed SNPs in LD with the causal SNP will each show only weak or moderate effects because each typed SNP serves as an imperfect surrogate for the causal SNP.

Therefore, it could be advantageous to consider the joint effect of multiple SNPs in analysis ^15^ because it is probable that several of these markers are in LD with the causal SNP and could capture the true effect more effectively than could analysis of individual SNPs. Finally, individual-SNP analysis considers only the average and/or marginal effect of each SNP and therefore fails to accommodate epistatic effects even when in LD with the causal genes. Epistatic interactions between SNPs can contribute to disease susceptibility such that individual SNPs may show little individual effect but their joint interactions may have a much larger effect. Even exhaustive analysis of individual SNPs will not be able to detect such effects because the large number of potential interactions exceeds the statistical power of even extremely large samples ^16^. As an alternative strategy for analysis, it has been proposed to group SNPs together into SNP sets along the genome and to perform genome-wide tests for SNP sets instead of individual SNPs. SNP-set-based analysis uses information from multiple correlated SNPs that are grouped on the basis of prior biological knowledge. As a result analysis of SNP sets has the possibility to provide improved reproducibility, interpretability, and increased power, especially when the effects of individual SNPs are weak or moderate ^17-19^.

There have been several earlier attempts to describe multi-marker tests (that is, tests of multiple SNPs and/or other biological markers) to overcome the limitations of tests based on individual SNPs. The first class of multi-SNP test was based on individual-SNP analysis using the most significant p-value as the p-value for the set of loci, and then correcting for having done multiple tests ^18,20-23^. However, such tests still rely strongly on individual-SNP analysis, and when the individual SNPs are not in high LD with the causal variant, they may have low power and they cannot accommodate complex genetic effects and interactions.

Likewise extension of multi-marker tests for multiple SNPs or haplotypes via multivariate regression ^24^ often offer little benefit over methods based on individual-SNP analysis because they have a large number of degrees of freedom ^25^. It has been proposed to compare pairwise genetic similarity with pairwise trait similarity to solve the problem of many degrees of freedom ^26-28^. However, these tests still have major limitations: they assume that all variants have the same direction of effect, i.e., all the minor alleles for each SNP increase risk or all minor alleles decrease risk^28^, or require expensive permutation analyses that may be impractical for some GWAS settings, or do not allow for easy covariate adjustment ^11^.

A second class of multi-marker tests consists of methods try to leverage explicit population-genetics models to pinpoint the causal locus. Many involve reconstructing the sample phylogeny to guide the analysis and infer the causal mutation ^30, 31^. If the population-genetics model assumed is realistic and correct, such problem-specific methods should have high power. However, it is difficult to validate the assumed models, and most procedures are computationally intensive, such that in real applications the models need to be simplified. Once again, these models usually fail to allow for covariate adjustment. Computational efficiency and ease of covariate adjustment give a practical advantage to the logistic kernel-machine regression test over population-genetic modeling.

An alternative analytical strategy was proposed by Dr Lin. She proposed to group SNPs together into SNP sets on the basis of proximity to genomic features such as genes or haplotype blocks, and then to test the joint effect of each SNP set. Testing of each SNP set uses the logistic kernel-machine-based test^29^, which is based on a statistical framework that allows for flexible modeling of epistatic and nonlinear SNP effects. This flexibility and the ability to naturally adjust for covariate effects are important features of our own test, which is an extension of Lin's approach that give it major advantages over individual SNP tests and existing multimarker tests. The logic behind our extension of Lin's analysis strategy is that we can extract information found in the joint relations of multiple SNPs to improve the power to detect true effects.

Consequently, the choice of the basis for grouping multiple SNPs can influence the power of the approach. Lin and her colleagues focused on grouping SNPs on the basis of their proximity to a known gene and noted that this allowed them to reduce multiple comparisons and to harness local LD structure in order to improve the power for capturing untyped SNPs. Using genes as the genomic features of interest allowed them ^11, 12^ to map approximately 310K SNPs to 18K SNP sets.

However, it may be that the causal SNP lies far from a known gene, in which case groupings based on genes (and, by extension, pathways) will fail to capture the effect of interest. To augment coverage of gene-desert regions of the SNP sets identified by Lin and others ^11, 12^, SNPs can be grouped on the basis of additional genomic features, such as evolutionarily conserved regions. Such groupings again may allow us to harness local correlation. A moving window approach will be useful for capturing all genotyped SNPs, but direct interpretation of SNP-set analysis results are more difficult. Groupings via haplotype blocks are attractive because they make explicit use of the LD information. Use of haplotype blocks will allow for comprehensive coverage of the entire genome and will remove the need to explicitly predefine genomic features of interest. Beyond harnessing local LD structure to boost power, another important feature of Dr Lin’s approach is the ability to model the joint effect of multiple, independent, causal signals as well as possible epistatic effects.

Practically, however, finding a SNP-set formation strategy that optimize this approach can be difficult. It was suggested in ^11^ that using a gene or moving-window strategy can certainly capture multi-SNP and epistatic effects among SNPs that are located close to one another on the genome, but identification of such signals among SNPs that are distantly placed will not be possible. Indeed they suggested that a potential strategy would be to use prior biological knowledge. In particular, WU et al pointed out that if multiple SNPs are expected to affect the disease risk, it is reasonable to expect them to lie within genes in the same pathway or in genes with similar function; hence, forming SNP sets on the basis of pathways can potentially capture such effects. Unfortunately, WU et al concluded that a systematic approach for identifying such grouping structures at the genome-wide level is not obvious and that to avoid bias in our testing procedure, any grouping strategy must be made without consideration of the case-control status of the subjects in the data set. Wu et al said that groupings must be made with the use of information from external sources, prior studies, or unsupervised statistical methods ^11, 12^, and that SNP-set formation strategies will improve with advances in our knowledge of the genome and genomic structures.

**5. The PGMRA method: Summary**

Our approach implemented the suggestions that emerged from Dr Lin’s work in order to uncover sets of multiple SNPs that may be correlated even at long distances on the same chromosome or on different chromosomes. We extended the approach in ^11, 12^ by using an “unsupervised statistical method” termed Phenotype-Genotype Many-to-many Relations Analysis (PGMRA, Figure S1). However, we used a purely data-driven method without biasing the search by using external sources, prior studies, or knowledge of the genome and genomic structures, such as genes, pathways, or additional genomic features, such as evolutionarily conserved regions. Indeed, our SNP sets can share SNPs but not subjects, as expected because they involve the same SNPs but with different allele values (both alleles of a SNP can act as risk alleles in different genetic contexts) in different subjects ^7^. Each SNP set was composed of a particular group of subjects described by a particular set of homozygotic and/or heterozygotic alleles; subjects and/or SNPs may be present in more than one set ^1, 32, 33^. These SNP sets and their relations with one another characterize the genetic architecture of disease-associated SNPs in all subjects, including cases and controls.

Given a genotype database from a GWAS represented as a matrix of [SNPs x subjects] and a corresponding phenotype represented as a matrix of [features x subjects], the method for dissecting the architecture of a disease is composed of 6 steps. These steps are described for example elsewhere ^7^, where a SNP set is a sub-matrix or bicluster ^1^ harboring subjects described by a set of SNPs sharing similar allele values ^1, 11^, and the features are the TCI subscales. More generally, PGMRA uses a Generalized Factorization Method (GFM) to dissect a GWAS into SNP sets ^1, 11^ based on the Fuzzy Nonnegative Matrix Factorization method (FNMF) ^1^ algorithm (Figure S1B-C). FNMF is based on the bioNMF method ^34^, and uses it as a default basic factorization method (Figure S1D). FNMF allows detection of outliers and overlap among sub-matrices ^1^. The GFM applies FNMF recurrently to generate multiple matrix partitions in each domain of knowledge (genotype and phenotype) using various initializations with different maximum numbers of sub-matrices *k* (where $2\leq k\leq\sqrt{n}$and *n* is the number of subjects), and thus avoids any assumptions about the ideal number of sub-matrices.

Notably, the PGMRA method identifies local partitions of datasets, which provides substantial advantages over classical clustering approaches. Averaging and comparing groups would be expected to miss real differences if such differences are localized in different locations, that is, in different subgroups of people with distinctive features (Figure S1F). In contrast to classical clustering techniques, such as hierarchical clustering ^35^ and k-means clustering ^36^, we used biclustering techniques that do not require subjects in the same bicluster to perform similarly over all features exhibiting changes. Classical clustering methods derive a global model whereas biclustering algorithms produce a local model in which signals emerge only in particularly relevant dimensions.

To guarantee that sub-matrices converge to the same solution and, given the non-deterministic nature of NMF and its dependence on the initialization of the *W* and *H* vectors, PGMRA runs the analysis 40 times for any *k* maximum number of allowed submatrices with different random initializations of the vectors to select those that that best approximate the input matrix ^37^. To estimate the precision of sample statistics of the SNP sets (variance of the *W* and *H* vectors) we use a leave-one-out technique (jackknifing) 1000 times on the SNP domain, which produces more than 90% support for all identified sets with an average variance of approximately ±5% of their corresponding *W* and *H* vectors ^34^. Finally, we modified the sampling technique to ensure the occurrence of the remaining sets after a leave-one-set-out procedure ^38^. This sampling and estimation method was applied in the current study sample reported here with more than 90% of support.

By incorporating *a posteriori* the status of the subjects (see Table S1) to SNP and phenotypic sets, the method is able to calculate the well-being and ill-being probabilities of such sets and their associations (see ***eqn. 5***, Map a disease risk in ^7^). For example, as noted previously, the well-being status was defined by the top decile of *SDxCOxST* and the ill-being status was defined by the bottom decile of *SD+*CO based on independent prior empirical wor*k*. *Q* is the weights given by epidemiologic risk of a disease in each SNP or phenotypic set (e.g., 0.01, 0.1 and 1 for cases, relatives and controls, respectively) ^1^, which here is ignored since subjects were from the general population and so generally considered more-or-less healthy. Once this procedure occurs, the method becomes semi-supervised, and posterior statistical significance of the SNP sets, and phenotype sets can be calculated using kernel-based and multivariate statistical analysis ^1, 11^. We used conservative risk estimation for the associations between a SNP set and a clinical, which was calculated as the maximum value of both sets.

PGMRA co-clusters SNP sets with phenotype sets into associations calculating the probability of intersected subjects using coincident tests based on hypergeometric statistics (PI_hyp_, ^33, 39^, equivalent to Fisher's exact test). The significance of the genotypic-phenotypic associations was tested by generating a permutation test, as described below and in ^1, 7, 40^. All optimal relations had empirical p-value ≤ value < 5E-03. The probability of well- or ill-health for an association is calculated by the maximum probability of each related set (i.e., most conservative risk evaluation). These associations are organized into multilocus networks connected by sharing subjects and/or features (e.g., SNPs, symptoms), where shared SNPs between two SNP sets may differ in the allele values and have distinct genomic consequences ^7, 19, 41^. This framework constitutes a knowledge base and characterizes the architecture of the phenotype. Further methodological descriptions of PGMRA are available in ^1, 32, 38, 42-45^, and its web server application is online at <http://phop.ugr.es/fenogeno> ^1^. Fast parallel software implementations were run at the Center for High Performance Computing (CHPC) facility at WUSM.

**6. The PGMRA algorithm**

PGMRA uncovers a deep architecture (Figure S1A) containing multiple sub-networks each uncovered by the NMF method used as a deep autoencoder^46^ (Figure S1D) in a particular domain of knowledge (genetics, clinical symptoms, TCI, voxels in neuroimages). Our implementation of the NMF, termed Fuzzy Nonnegative Matrix Factorization method (FNMF), learns and is optimized as described below and elsewhere^1, 7^. The nodes of one sub-network learned in a particular domain of knowledge (e.g., genetics) by a deep FNMF autoencoder (consensus clustering, see above) are connected by shared subjects and/or features (SNPs in genetics, subscales in TCI). Two sub-networks, learned from different domains of knowledge (e.g., genetics and TCI), are assembled by calculating the probability of intersection of their nodes and selecting those optimal connections based on multiobjective and multimodal optimization techniques (see above). These sub-networks constitute the pooling set of deeper layers of the network^47, 48^. Overall, the full network integrates different domains of knowledge into interpretable associative networks. The method utilized in this manuscript and described in (Figure S1B-C) is unsupervised because we want to extract new knowledge. However, it can be easily extended to a semi-supervised approach by adding a labeling and a classification layer ^49^ (Figure S1D). It should be noted that each layer has its own learning process (see below) and, instead of the weights in a neural network model, their outputs are interpretable relationships (Figure S1A,E).

Given a genotype database from a GWAS represented as a matrix [SNPs x subjects], the full method for dissecting the architecture of a disease is composed of 8 steps (Figure S1), where a SNP set is a sub-matrix ^1^, here also termed biclusters ^37, 50^, comprised of a subgroup of subjects described by a particular subgroup of SNPs sharing distinct allele values ^1, 11^. In the current study, the phenotype database is composed of TCI subscales of character [TCI scores x subjects], and its analysis is approached in the same way as described for the genotype. Genotypic sets are independently learned from the phenotypic set, but *a posteriori* associated into relationships.

As a convenient guide for readers, we will describe each of the steps in the PGMRA analysis as implemented in the PGMRA webserver and illustrated in Figure S1 in the following 8 sections (i to viii).

**(i) Identify optimal genotypic and/or phenotypic sets**

*Mathematical description of the NMF* (Figure S1D,E): We consider a dataset consisting of a collection of n subject samples, which we use to characterize a domain of genotypic (SNPs) or phenotypic (TCI) states of interest. Here, we illustrate the NMF by the genotypic type of data, but can be extended to any other type of data. The data are represented as an *m* x *n* matrix X, whose rows contain either the allele values of the *m* SNPs in the *n* subject samples. Using the NMF, we find a manageable number of factors *k*, positive local and linear combinations of the *n* subjects and the *m* SNPs, which can be used to distinguish the genetic profiles of the subtypes contained in the dataset. Mathematically, this corresponds to finding an approximate factoring, *X_mxn_* ~ *W_mxk_* x *H_kxn_*, where both matrices have only positive entries and hence are biomedicalli meaningful ^1, 37, 51, 52^. *W* is an *m* x *k* matrix that defines decomposition model whose columns specify how much each of the subjects contributes to each of the *k* factors. *H* is a *k* x *n* matrix whose entries represent the SNP allele values of the *k* factors for each of the *n* subject samples. In our implementation either a subject or SNP can belong to more than one factor ^1, 32, 33^.

*Transforming NMF k factors into sub-matrices or biclusters which are interpreted as SNP or phenotypic sets*: The original bioNMF method ^37, 50 , 53^ uses the non-smooth variant of the NMF algorithm (nsNMF). This variant achieves an easier interpretation of the factors (*k*) due to the intuitive sparse, non-overlapped part-based representation of the data. Once the *W* and *H* matrices are calculated, the method selects the most representative features and observations (subjects) for each factor in order to build the biclusters. The bioNMF algorithm defines the factor-specific rows or columns as those rows or columns in the *H* and *W* matrices, respectively, that show high coefficients for a given factor, as well as low coefficients for the other factors. Given a certain factor *k*, i.e., the lth column of *W*, all features in the dataset can be properly sorted by their association to the local pattern captured by this factor (Figure S1D(ii-iii)). At the same time, observations/subjects can also be sorted by their coefficients in the corresponding factor, that is, the lth row of H. This operation is carried out in one-to-one correspondence among columns of *W* and rows of *H*, generating *k* natural ordinations of the matrix in which features and subjects highly related in a sub-portion of the data. The set of selected rows and columns for each factor define a bicluster.

We developed a fuzzy variation of the bioNMF biclustering method named Fuzzy NMF or FNMF ^1^, where every column or row can belong to many biclusters or, eventually, to all of them ^32, 33, 38^. In addition, our FNMF includes a strategy to identify and discard outliers from the biclusters, as in a possibilistic clustering method ^38, 54, 55^. Unlike the bioNMF biclustering method, our FNMF analyses each factor by selecting the rows or columns with the highest values based on a threshold established as an input parameter. This threshold indicates the level of fuzziness, and in turn, which values will belong to a bicluster. The threshold is defined in the unit interval [0-1]. For example, the threshold for factor *i* in the matrix *H* is calculated as:

*
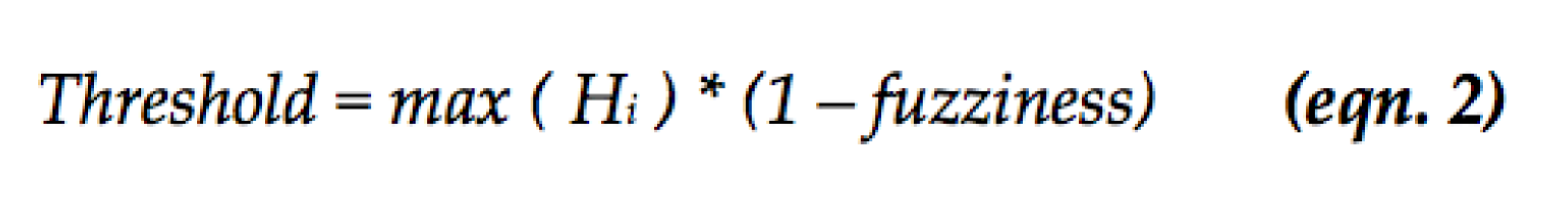
*

where all values above the threshold will be kept in the bicluster. The selection process for one factor takes into account only the values within that factor (that is, they are independent of the values of the rest of the matrix, Figure S1D(iii)). The fuzziness allowed in the current study was 30%. From now on, we will use the terms sub-matrix, bicluster, or set (SNP or phenotypic) as synonyms that serve to emphasize specific features of the same thing in different analytical or clinical contexts.

*Decomposing the data into a multilevel family of sub-matrices* (Figure S1E): The Generalized Factorization Method (GFM) applies a basic factorization method recurrently to generate multiple matrix partitions using various initializations with different maximum numbers of sub-matrices *k* (e.g., $2 \leq k \leq\sqrt{n}$ where *n* is the number of subjects), and thus, avoids any assumption about the ideal number of sub-matrices (see ^1^ for a rationale about the use of unconstrained number of sub-matrices or clusters). Specifically, we use FNMF as described in the prior section. For each run of the basic factorization method ($2 \leq k \leq\sqrt{n}$), all sub-matrices are selected to compose a family of genotypic SNP sets *G_k* ={G_*k_i*}, where $1 \leq i \leq k$. Each *G_k* family, as well as all families together *G* ={G_*k*} for all *k*, may include submatrices (i.e., sets) that are overlapping, partially redundant, and different in size.

*Learning the W and H matrices of FNMF:* Due to the non-deterministic nature of FNMF, it may not converge to the same solution on each run because of the random initial conditions used. Therefore, we execute the algorithm 40 times, as was originally suggested for the bioNMF algorithm ^1^, with different random initializations for selecting the *W* and *H* matrices that best approximate the input matrix. FNMF makes use of the convergence method described in ^56, 57^ to establish the stopping threshold that controls the algorithm convergence on each run. Each 10 iterations, a connectivity matrix *M* of size *C* × *C* is computed, where *C* is the number of columns of matrix *H*. Each entry *M_ij_* in this matrix is set to 1 if column *i* and *j* in *H* have their maximum value for the same factor (i.e. on the same row in *H*), and 0 otherwise. If the connectivity matrix stops changing after a certain number of iterations (which equals the stopping threshold multiplied by 10), the matrices are considered as having converged and the algorithm stops the current run. The learning process of the *W* and *H* matrices is performed with projected gradient descent methods ^56, 58^.

*Optimally assembling the families of sub-matrices*. Because sub-matrices can be defined at different levels of granularity, we apply a competitive learning approach (i.e., consensus clustering ^59 , 60^) to select and assembly optimal, non-redundant, and cohesive sub-matrices using multiobjective and multimodal optimization techniques. Optimal sub-matrices were obtained as a tradeoff between two opposing objectives: sensitivity and generality ^32, 33, 45, 61-63^. Sensitive sub-matrices tend to be composed of few observations (i.e., subjects) described by multiple features, whereas specific sub-matrices are composed of many observations described by few features. A Pareto-optimization strategy searches for solutions that are non-dominated in the sense that there are no other solutions superior in all objectives being selected (i.e., close to the Minimum Description-Length (MDL) ^64^). The dominance relationship as a minimization problem is defined as:


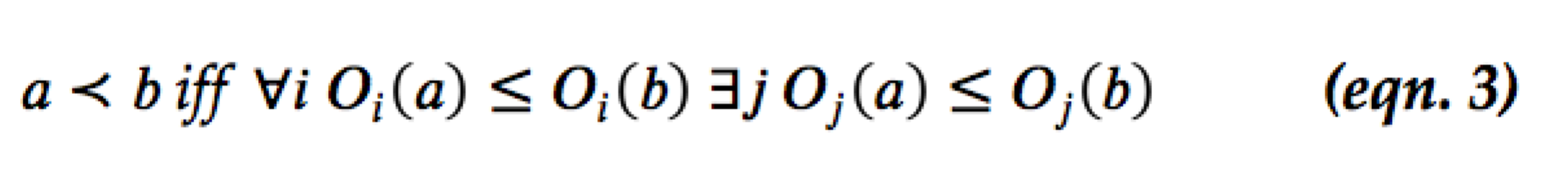


where the *O_i_* and *O_j_* are either specificity or generality objectives. Optimization of small sets of sub-matrices was exhaustively implemented, whereas evaluation of large sets is approached by Genetic Algorithms, as described in ^38, 44, 65^. Another indirect objective considered for the evaluation of sub-matrices is the generation of diverse patterns that completely describe objects (subjects). Therefore, our approach evaluates the sensitivity and generality objectives described above in a local niche ^33, 61-63^. Both sensitivity and specificity measurements are based on counting objects within a sub-matrix without distinguishing among them (e.g., # subjects). However, diversity differentiates which objects are within a sub-matrix, and thus, sub-matrices harboring distinct objects are allocated in different niches. These niches are calculated using Jaccard’s metric between sub-matrices ^44, 65^ (i.e., inclusion of subjects):


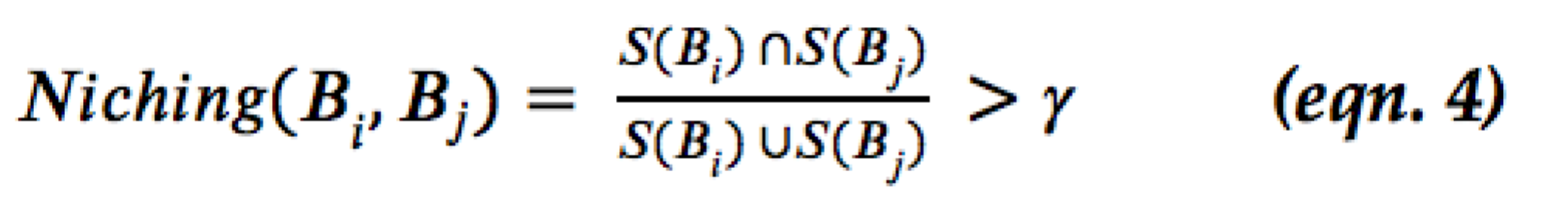


where *B_i_* and *B_j_* were two different sub-matrices, the *S* functional retrieves the subjects in the sub-matrices in a particular niche, and $\gamma$ is the size of the niche determined by the degree of overlap (i.e., intersection) between sub-matrices. Here the assumption is that the niches are equivalence classes dictated by the degree of overlap/inclusion between subjects in the sub-matrices. In sum, sub-matrices compete with each other if and only if they are in the same niche. For example, given two sub-matrices where one of them has the same or even worst sensitivity and generality than the other but correspond to different sets of subjects, both sub-matrices will be preserved because they are in different niches.

**(ii) Perform a statistical analysis of SNP sets (accessed via the PGMRA web server ^1^)**.

Use the R-project package SKAT ^11, 12^ to evaluate the significance of each SNP set. We used identity-by-state (IBS) as a kernel because the analyzed variants are not rare but common, and therefore using the “weighted IBS” kernel would not be adequate ^11, 12^. Since the SNP sets can overlap, we run each one separately. The gender and ancestry (3 PCs) of the subjects were used as covariates (see above), and the default remaining parameters were utilized. To run the SKAT test, we transformed the unsupervised SNP sets into a supervised form by labeling their subjects *a posteriori*. The ***global labeling*** assigns to each subject their case/control status, which in this case is the well-being or ill-being status. Then, the SKAT method evaluates the ability of each SNP set to classify the status of all subjects based on the SNPs included in such set. Another approach is termed ***local labeling***, which for each SNP set tags the subject within and outside that set with two different labels. Then, the SKAT method evaluates the probability of each SNP set to differentiate from the other sets. This is done because we hypothesize that all optimal SNPs are required to explain the distributed heritability ^7^.

**(iii) Calculate the frequency of the subject status within a SNP set**

Once the health status of the subjects is incorporated *a posteriori*, the frequency of that status is calculated as a weighted average function of its observed epidemiological occurrence among all subjects in a particular SNP set. Here, the probability of well- or ill-being status is defined as:


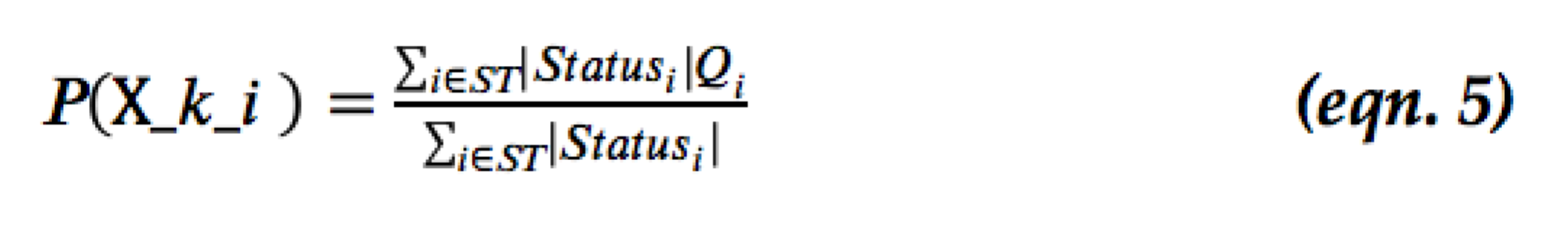


where X_*k*_*i* is a SNP or phenotypic set, *Status* is the status of the instances, and *Q* is the weights given by the observed epidemiological risk of that status in each SNP set (e.g., 0 and 1 for controls and cases; 0.01, 0.1 and 1 for cases, relatives and controls, respectively) ^1^. The frequency of the status for all SNP sets is interpolated as a surface using the *tgp* and *latticeExtra* packages in R-project, respectively.

**(iv) Discover and encode relations among SNP sets into topologically organized networks**

Co-cluster all SNP sets by calculating the pairwise probability of intersection among them using the Hypergeometric statistics ^33, 39^ (PI_hyp_) on intersected SNPs: PI_hyp_ (G_*e*_*q,* G_*r*_*w*) (see below, ***eqn 6***), where *q* and *w* are SNP sets generated in runs with a maximum of *e* and *r* number of sub-matrices with the FNMF method. Two types of intersections are evaluated: SNPs and/or subjects. Connected and disjoint associations are organized into multilocus networks connected by sharing observations (subjects) and/or features (SNPs, symptoms), where SNPs shared by two SNP sets may differ in the allele values and have distinct genomic consequences ^7^.

Clearly, single nodes (e.g., G_19_5 in Figure 1B) or nodes connected only to one another in a non-cycling path are disjoint networks. Other sub-networks or sub-graphs are visualized as different entities but still connected by a few edges. We searched for sub-networks of SNP sets composed of connected nodes. Links were recognized when sets shared 25% or more of their SNPs (red lines in Figure 1B) or of their subjects (blue lines in Figure 1B). Colloquially speaking, a clique of people is defined as a group with shared features, such as individuals who share many activities with one another often and not much with others. Likewise a clique of SNP sets is a group of SNP sets that are highly connected with one another and not with other SNP sets. Here we defined a subnetwork as a sub-graph with more than four 3-vertex cliques ^66-68^ where a clique was specified as a triad of adjacent nodes that were fully connected. In general, cliques connected by other nodes belong to the same sub-network. Cliques connected by a single edge belong to different sub-networks. Nodes connected to a clique by one edge or a succession of non-cycling edges (i.e., Hamiltonian paths ^69^) belong to the same sub-network as that of the clique. For example, one network in Figure 1B is composed by [G_3_1, G_8_8, G_7_2] , [G_8_8, G_21_3, G_7_2], [G_8_8, G_3_1, G_21_3], [G_3_1, G_21_3, G_7_2], where [G_7_2, G_33_15] and [G_7_2, G_16_9] are Hamiltonian paths included in the sub-network. Furthermore, [G_12_8, G_11_4, G_8_8] also belong to the same network since it is connected by G_8_8. We described such subnetworks that are highly internally connected as relatively disjoint from the other components of the displayed architecture.

**(v) Identify optimal and significant phenotypic sets (Implemented in the PGMRA web server ^1^).**

Next, we created a phenotype database by collecting the TCI character measurements at the sub-scale level encoded in the Likert scale. For efficiency and interpretability, in our case, each sub-scale variable was decomposed into two variables: the original variable *x* and the complementary *Lx* (Low x), which is 5-*x* in the Likert scale. The phenotype data was codified in a [phenotype features x subjects] matrix, where the columns and rows correspond to subjects and phenotypic features, respectively. To identify phenotype sets (as implemented in the PGMRA web server ^1^) we apply the FNMF method with the phenotype database–instead of genotype database— as described above in Step 1, where a phenotypic set is a sub-matrix ^1^ harboring subjects described by a set of phenotypic features sharing similar values (i.e., *P_h_j*, where *j* is a phenotypic set generated in a run with a maximum of *h* number of sub-matrices). To select the optimal phenotypic sets, we applied the competitive learning process to the phenotypic sub-matrices as described above in Step 2. The statistical evaluation of the phenotypic sets was performed in a fashion similar to that performed on the genotype. However, the SKAT test was replaced by a Chi-square test that evaluates a logistic regression with respect to the null model to get a p-value that reflects the ability of a phenotypic set to discriminate subjects by their status (see *lrm* function of the R-package *rms*). To encode the phenotypic sets into a manageable set of profiles or superclusters, we applied the FNMF method recurrently as a typical deep learning strategy (Figure S1E).

Superclusters associate subsets of phenotypic sets, here termed character sets, at a lower level of granularity (i.e., detail) and represent semantic profiles that facilitate the communication of results without reducing the diversity of phenotypic sets encoded in such subsets. These superclusters are calculated by recurrently applying FNMF to the matrix encoding [phenotypic sets x TCI subscales]. The optimal number of sub-matrices was selected by the Cophenetic index ^50^ (see below deep NMF section, Figure S1E).

**(vi) Identify optimal genotype-phenotype latent architectures**

To identify genotypic-phenotypic relations, we co-clustered SNP sets with phenotypic sets into relations using the Hypergeometric statistics (PI_hyp_, see below, ***eqn 6***) on intersected subjects, where *R_i,j_* = PI_hyp_ (*G_k_i*, *P_h_j*), *G_k_i* and *P_h_j* are SNP and phenotypic sets, respectively, and *p* is the intersection of subjects. Relations *R_i,j_* < *T* constitute the genotypic-phenotypic architecture of a disease. The significance of the relations (*T*) was established by the p-value provided by the Hypergeometric-based test ^33, 39^.

The degree of overlap between two sets (SNP and/or phenotypic) was assessed by calculating the pairwise probability of intersection among them based on the Hypergeometric distribution ^33, 39^ (PI_hyp_):

***
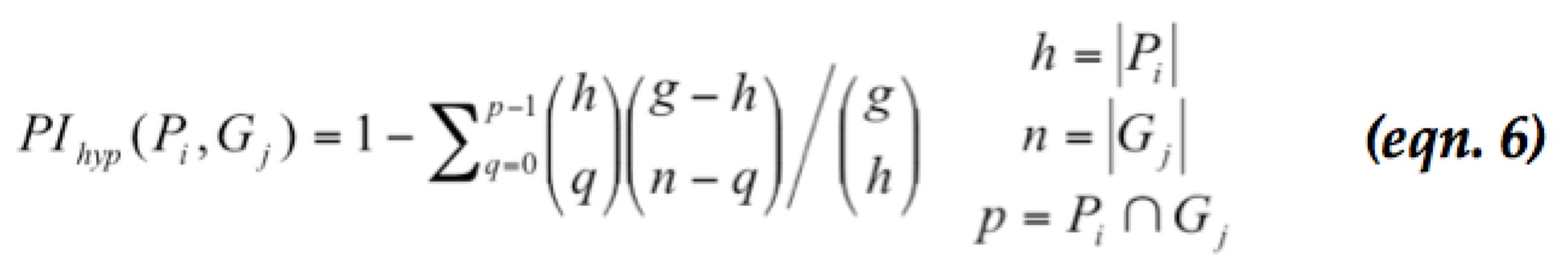
***

where *p* observations belong to a set *P_i_* of size *h*, and also belong to a set *G_j_* of size *n*; and *g* is the total number of observations.  Therefore, the lower the PI_hyp,_ the higher the overlap . The (p-value of) Hypergeometric “test” is used here as a measure of association strength. The real test (p-value) of genotypic-phenotypic relationship was provided through the permutation procedure.

The permutation test designed to evaluate genotypic-phenotypic relations was implemented as follows. Statistical significance values were obtained by 1,000 independent permutations due to the comparisons between all possible generated SNP sets (i.e., 1034, from 2 to $\sqrt{sqrt(n})$), and possibly overlapping SNP sets were identified here as follows ^1, 40^: a) assign random subjects to a genotypic cluster (set) of random size; b) assign random subjects to a phenotypic cluster of random size; c) calculate the PI_hyp_ between the two clusters and accumulate the value ^39^. These values form an empirical null distribution of PI_hyp_ used to calculate the empirical p-value of an identified relation. All optimal relations had empirical p-value ≤ 5.00E-03. (Note: the permutation test accounts for multiple comparisons ^70^.)

**(vii) Semi-supervised learning: transforming unsupervised knowledge bases into supervised classifiers**

*PGMRA semi-supervised classifier*. PGMRA is an unsupervised method developed to identify new knowledge –instead of reproducing the available supervised knowledge. This is a key difference between unsupervised and supervised (classification/regressions) learning. Here, we combine both types of learning in what is called a semi-supervised approach (Figure S1A, D(iv)). Once the new knowledge is learned, it is used to label the observations and utilized for classification. To implement the PGMRA Bicluster classifier based on biclusters we first integrated all selected optimal biclusters in a single matrix. This matrix contain all features identified by any of the optimal biclusters as columns and all subjects as rows. Second, we assigned labels to each subject based on their status. For example, in the case of the genotypic database, we can assign the phenotypic status such as case-control or well- or ill-being values to predict these outputs, which are also the targets of the global version of the statistical test SKAT. Indeed, the status can be the SNP set or the sub-network label, which are also the targets of the local version of the statistical test SKAT. The resulting matrix encodes the fuzziness intrinsic to biclusters defined at different levels of granularity, as well as observations/subjects labeled with more than one status. Another way of using a classifier is to consider the frequency of the status of each SNP set (e.g., epidemiological risk), and thus, behaving as a continuous regression ^1^.

*kNN classifier*. Once the integrated matrix is developed, we applied the k-Nearest Neighbors (kNN) classifier to show that even a rudimentary classifier works well with structured data such as those pooled from the optimal biclusters (PGMRA also used Decision Trees ^71^. Given the similarity metric between the training observations and a new instance to be classified, kNN assigns the most common label among the ((k)) closest neighbors of the training data set ^71^. We used the Euclidean distance as a metric, adjusted the dataset for imbalanced observations (see below), calculated the best k from 1 to √n, where n is the number of observations, and utilized a sampling technique in conjunction with distinct metrics to validate the obtained results (see below).

*k-Fold Cross Validation*. The goal of cross-validation is to test the model’s ability to predict new data that were not used in estimating it, in order to flag problems like overfitting and to give an insight on how the model will generalize to an independent dataset. The *k*-Fold Cross Validation is an iterative procedure where the data are divided into k equal folds in each iteration, where one fold is used for validation and all the others for training the classifier. We used stratified *k*-fold cross-validation, where all folds contain a proportional number of examples of each class. The process is repeated *k* times and the average of the *k* validation folds are used as a performance measurement. We used the standard choice of 5-fold for imbalanced data ^72, 73^.

*Metrics.* The sensitivity or true positive rate (TPR) is defined as the proportion of true positives (TP) detected by the classifier divided by the total true positive (TP) and false negatives (FN) examples (TPR = TP / (TP + FN)). The specificity or true negative rate (TNR) is defined as the proportion of true negatives (TN) examples correctly predicted by the classifier divided by the total TN and false positive (FP) examples (TNR = TN / TN + FP). The false positive rate (FPR) consists of the errors of the classifier when it incorrectly predicts the positive class (FPR = 1 – specificity). The accuracy is defined as (TP + TN) / (TP + FP + TN + FN). We also utilized area under the curve (AUC) of Receiver Operating Characteristics (ROC), where the ROC is the rate of TPR vs FPR at different classification thresholds ^74^.

*Imbalanced datasets*. Oversampling and undersampling in data analysis are techniques used to adjust the class distribution of a data set (i.e. the ratio between the different classes/categories represented). Because our dataset is imbalanced with respect to the well- and ill- being status, we used one of the most typical techniques to compensate for imbalance; this technique is the Synthetic Minority Over-sampling Technique (SMOTE, ^72, 73^). To create a synthetic data point, SMOTE takes a sample from the dataset, and considers its *k* nearest neighbors (in feature space). Then, it calculates the vector between one of those *k* neighbors, and the current data point. Then this vector is multiplied by a random number *x* which lies between 0 and 1. Add this to the current data point to create the new, synthetic data point. In this work, we combined oversampling and undersampling techniques into a hybrid strategy.

**(viii) Bioinformatics analysis of the SNP set-related genes, their molecular consequences, and pathways**

For each SNP set, we analyzed all genes in the cluster, including the location of the SNP with respect to the gene, the type and number of genes comprising each SNP (e.g., distinguishing protein-coding genes, ncRNA genes, pseudogenes, and regulatory genes), the possible transcripts affected and the position where they are affected (e.g. coding region, distance to stop codon, splicing site, intron, UTR, etc.), and finally annotations about promoter and intergenic regions were inspected. All possible molecular consequences of each SNP in the function of the gene were considered in the analysis. A detailed analysis of SNPs and mapped genes can reveal at least three complex scenarios affecting multiple genes in different fashions (e.g. activation, repression, antisense modulation) and producing different molecular consequences, which were considered in queries of the Ensemble version 88 and NCBI databases (Entrezgene, Protein, Unigene). We evaluated whether a single SNP within a SNP set could produce different consequences in affected transcripts, whether multiple SNPs within a SNP set can jointly affect one or more genes in different ways, and finally whether multiple SNPs within different SNP sets can distinctively affect the same gene.

We investigated the regulatory and protein domain binding regions *de novo* using information available in known transcription factor databases and the sequences of the identified genes as inputs for our predictive bioinformatics algorithms ^32, 33, 38, 44, 75-81^, which perform novel predictions on genetic networks, RNA genes, and protein-protein interactions. Long non-coding RNA genes (lncRNA) were considered in particular because they were overrepresented among genes associated jointly with temperament and character. Omic data, including annotations about individual genes and families of genes were obtained from the Haploreg database ^82^, the Ensembl version 88, GeneALaCart, TRANSFAC ® release 2017.1, Pfam v30, and the NCBI web services. The RNomic analysis included the Linc2GO ^83^ and the LncVar ^84^ databases, and databases related to CircRNAs such as starBase v2.0, and circBase 0.1 ^85, 86^ in order to decode possible interactions among RNA genes. Once we obtained the information described above, we generated a list of relevant genes that were used to query the databases Nextbio, GeneALaCart v.4.5, DAVID v6.8, KEGG v82.0, Reactome v58, BioCyc v.21.0, WikiPathways, and Pathway Interaction Database ^87-90^ in order to identify pathways related to the genes. Overall, we found that the products of genes uncovered by the SNP sets are included in several well-known, relevant, and interconnected signaling pathways. Annotation information obtained from Haploreg v4.1 ^82^, Ensembl version 88, NCBI, and GeneALaCart v.4.5, and TRANSFAC web services was manually curated.

**7. Estimation of heritability**

We used a method recently proposed by the International Schizophrenia Consortium ^91, 92^ to evaluate the amount of phenotypic variance explained by our associated loci in distinct validation genetic studies.


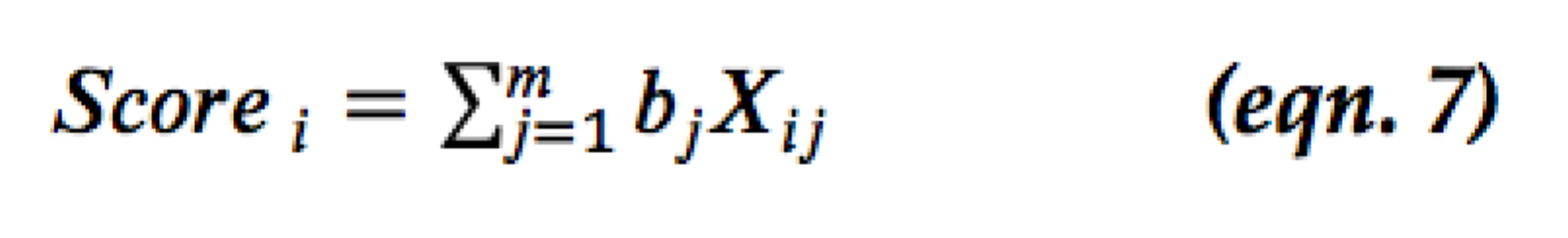


where *m*=number of SNPs, effect b of allele at locus *j*, and *X_ij_*  = number of reference alleles of individual *i* at locus *j*.

The method involved three steps of analysis: 1) selection of markers to build a prediction model, 2) scoring each individual based on the model and 3) estimation of variation explained using the scores as the predictor. First, we collected all SNPs identified by SNP sets that were associated with the empirical index of character in the Finnish sample. Second, using the selected SNPs we performed profile scoring for each individual calculating the correspondent *b_j_* for all SNPs using the regression model implemented in Plink. Third, the measure of variance explained (adjusted R^2^) was estimated from a linear regression model incorporating the score as the predictor and the empirical index of character as a single comprehensive phenotype for character as the outcome. The linear regression was calculated with the raw data and also after excluding outliers. The outliers were calculated by a trimmed regression analysis. This trimming approach is based on identifying all values that are at a specified distance from the regression line between the lowest and highest values for that sample. The trim value we used for deviation from the regression line was 5%.

The variance in the replicated samples was calculated in the same fashion. The initial set of SNPs is based on the union of the SNPs covered by the selected SNP sets in the Finnish sample and those covered by the corresponding SNP set homologs in a replication sample. Since one Finnish SNP set is covered by more than one set in the replica, we only used the best match. Most of the SNPs in the replications are included in the Finnish initial set of SNPs.

It can be argued that the heritability estimated by the R^2^ may tend to inflate the results. However, in our case, the possible inflation was compensated by the method tending to underestimate the heritability by its neglect of the complex genotypic-phenotypic architecture. This can be explained in fairly simple language for clarity in the following paragraph:

In order to calculate the heritability based on the trimmed linear regression approach, we utilized all SNPs identified by the 42 sets associated with character as if all SNPs had the same average effect on all subjects without taking into account their complex organization in sub-sets sharing only some subjects and/or some SNPs. This was necessary because at present there is no general solution for precise estimation of heritability for traits with a complex architecture, even when specified in detail as it is by the PGMRA method. The PGMRA method uses SNPs grouped in sets in which one set may cover some subjects and another set can cover a different group of subjects with some possible overlap. These two sets may have different sub-types of the phenotype, and thus, the subjects within each set are not associated with all the SNPs that are found when the SNPs from the two SNP sets are combined. Forcing the subjects to be associated with all SNPs from both groups reduces specificity and dilutes the strength of the association. Consequently, the R^2^ may tend to inflate the results, but the consideration of all SNPs together without the specificity of the SNP sets compensates for the inflation at least in part.

**8. Replicability of results**

**(i) Statistical replicability of sets of markers**

PGMRA has been applied to each one of the three samples here considered: Finnish, Korean and German datasets. Replicability has been evaluated at three levels: 1) SNP sets; 2) phenotypic sets; and 3) genotypic-phenotypic associations. SNP and character sets identified by PGMRA in the Finnish sample and replicated in the other samples were evaluated using Hypergeometric statistics and the corresponding empirical distribution (see above, ***eqn 6***) with a PI_hyp_ threshold <1E-05 and <1E-02, respectively. Replication of a SNP set and character association across samples considers the replication of 1), 2), and the strength of the association (PI_hyp_) of the homologous SNP and phenotypes sets in the target sample. Because all 3 measurements contributed to replicability, we selected the optimal replication associations by using multi-objective optimization techniques ^7^. This approach is based on selecting those associations that are non-dominated. One association is non-dominated by others if there is no association that is better in all objectives (1,2, and the strength of 3) than the non-dominated association (see above, ***eqn. 3***).

**(ii) Justification of the statistical replicability of sets of markers**

In probability theory and statistics, the hypergeometric distribution is a discrete probability distribution that describes the probability of successes (random draws for which the object drawn has a specified feature) in draws, without replacement, from a finite population of size that contains objects with that exact feature, wherein each draw is either a success or a failure. In contrast, the binomial distribution describes the probability of successes in draws with replacement. In statistics, the hypergeometric test uses the hypergeometric distribution to calculate the statistical significance of having drawn a specific success (out of total draws) from the aforementioned population. The test is often used to identify which sub-populations are over- or under-represented in a sample. This test has a wide range of applications in set theory and population studies ^1, 32, 39^.

The interpretation of the replication probability reflects the underlying stability of a group of observations or outcomes. In group theory and clustering in topology analysis ^1, 32, 39, 93^, this probability has been widely used to calculate stable clusters replicated by different methods, which helps to practically estimate the number of clusters in a data partition, which is a theoretically unsolved problem ^54, 55, 63^. Particularly in genetics, the probability of replication is used as an estimate of the probability that a gene set will be significantly expressed in a repeated study ^93^ or validated by sets of similar molecular information (co-clustering with enrichments ^39^, such as pathways and ontologies ^1, 32, 39^). Both the hypergeometric (Fisher’s exact test) and Wilcoxon tests are utilized to estimate the replication probability. The hypergeometric test takes the size of the overlap between a gene set and the list of differentially expressed genes as the test statistic, and reassigns labels without replacement (i.e. it keeps the marginal totals in the table constant). This is often used to calculate the significance for overrepresentation of the gene set among different sets of differentially expressed genes as a test for independence ^70^. Specific examples of enrichment in human genetics include higher replication rates and consistently stronger enrichment of eQTLs ^94^.

The hypergeometric analysis has served to logically focus replication efforts of SNPs that were found to be significantly associated in different samples or endophenotypes ^95^. In such applications the method assumes that a given phenotype is influenced at each quantitative-trait locus (QTL) by one or more causal variant(s), whose effect(s) can be approximated (or tagged) by a linear combination of multiple semi-independent observed variants at the locus. This linear combination of SNPs is termed a multi-SNP. For example, the problems of allelic heterogeneity and imperfect tagging of multi-SNP associations and the identification of significant multi-SNP associations has been successfully addressed with the hypergeometric test (^41^, better tan Yang et al. method). These examples and others ^1, 7, 96^ suggest that the hypergeometric test is a measurement that is useful for replicability analysis of sets of variables. Such analyses of sets of markers do not invalidate the use of meta-analysis of individual markers. A complementary alternative to the hypergeometric is the permutation test (see preceding section on Permutation test).

**(iii) Hierarchical and multi-omic replicability of markers**

Since reproducibility of individual genetic variants associated with risk across studies is low, we hypothesize that wherever individual genetic variants do not overlap, there may yet be a reproducible pattern in terms of functionally overlapping genes, families of proteins, or molecular pathways. Therefore, we tested the functional “semantic” replicability of the personality by including comparisons at different levels of granularity/omics to establish a multi-layer consensus of the architecture of the disease. The objective is to cast a wide net in order to capture those measurements that are informative, as well as identifying those that are not.

We systematically searched for genetic variants and corresponding genes that had previously been associated with personality as measured by the TCI (Supplementary Tables S20, S21). We used a proprietary text-mining tool developed in Ruby and Pearl script languages that automatically performed the following tasks and retrieved the results in an html format. First, the tool retrieved articles about human genetics containing TCI-related keywords from PubMed/NCBI. Second, we identified the genes related to each report and collected these genes in a list, which we called our reference list (NCBI). Third, we generated a list of the genes retrieved in the current studies of temperament and/or character traits, which we called our query list. Fourth, we compared the genes in the query list to those in the reference list to identify matches. Fifth, for those genes that did not match specifically in the first place, we retrieved the family of genes from Pfam/NCBI and checked if the genes in our query and reference lists matched at the family level of correspondence. Finally, for those genes that did not match in the first (i.e., specific gene) or second (gene family) level, we retrieved the pathways of the genes (from Entrez Gene, Ensembl, NextBio, DAVID, KEGG, Reactome, WikiPathways, BIOCYC), and performed comparisons at the pathway level. We evaluated the percentage of prior reports that we recovered as a test of the sensitivity and specificity of our method for identifying specific genes, particularly in consideration of the severe missing heritability problem encountered by prior GWAS of personality that had neglected genotypic-phenotypic complexity.

**9. Analysis of results**

**(i) QQ Plots and covariate analysis**

We tested the stratification effect by adjusting for gender and for the first 3 PCs (see preceding section on PCA analysis). Then, we calculated the lambda values and ran the QQ Plots (Figure S8). As expected, the experiment without covariates ($\text{λ}$ = 1.363) differs from that one that considers all the covariates ($\text{λ}$ = 1.094). It has been shown by different studies that the use of the first 3 PCs is usually sufficient to correct population stratification in GWAS studies ^9^.

**(ii) Statistical analysis: evaluation of SNP and phenotypic sets by additional tests**

The analysis of the SKAT that evaluates the ability of a SNP set to differentiate the well-being and the ill-being status of all subjects is presented in the main text (Table 1). In addition, as described in the Supplementary Methods, for each SNP set we tested the probability of finding its component subjects and SNPs together. In other words, we evaluated how well the SNPs contained in a SNP set distinguished their subjects from the other subjects outside the SNP set or how different is a particular SNP set from the others. 95% of the SNP sets associated with the Character phenotype exhibited a 5E-11 > p-value > 5E-77 (5% < 5E-07). These results strongly support the importance of each individual SNP set as a contributor to explain the total distributed heritability. Finally, we also evaluated the ability of the phenotypic sets to distinguish health of subjects (in terms of well-being versus) using a global logistic test. ~60% of the Character sets were significant discriminators of health status (1E-03 > p-value > 7E-120, and the remaining ~40% < 1E-02).

**(iii) Semi-Supervised approach: Classifying subject phenotype based on the genotype**

We applied a simple classifier to evaluate the advantage assembling the unsupervised learned knowledge into a single classifier, which still retained the structural knowledge of the biclusters. Because the well- and ill-being status distributions represent unbalanced data (1:10), we utilized the SMOTE algorithm as described above with 3% oversampling and 1.5% undersampling parameters to compensate for the imbalance. We apply 5-fold crossvalidation and obtained an average AUC of 0.928 and 0.932 for the well- and ill-being status in the Character phenotype, respectively (Figure S9). The accuracy with this phenotype varies in the interval [0.913, 0.934] for the well-being measurement, where the two best *k* values for the kNN algorithm were 1 and 15. The accuracy with the Character phenotype varies in the interval [0.912, 0.945] for the ill-being measurement where the two best *k* values for the kNN algorithm were 1 and 31.

**B. References for Supplementary Information**

1. Arnedo J, del Val C, de Erausquin GA, Romero-Zaliz R, Svrakic D, Cloninger CR *et al.* PGMRA: a web server for (phenotype x genotype) many-to-many relation analysis in GWAS. *Nucleic Acids Res* 2013; **41**(Web Server issue)**:** W142-149.

2. Raitakari OT, Juonala M, Ronnemaa T, Keltikangas-Jarvinen L, Rasanen L, Pietikainen M *et al.* Cohort profile: the cardiovascular risk in Young Finns Study. *Int J Epidemiol* 2008; **37**(6)**:** 1220-1226.

3. Ravaja N, Keltikangas-Jarvinen K. Cloninger's temperament and character dimensions in young adulthood and their relation to characteristics of parental alcohol use and smoking. *J Stud Alcohol* 2001; **62**(1)**:** 98-104.

4. Brandstrom S, Richter J, Nylander P-O. Further development of the Temperament and Character Inventory. *Psychological Reports* 2003; **93:** 995-1002.

5. Sung SM, Kim JH, Yang E, Abrams KY, Lyoo IK. Reliability and validity of the Korean version of the Temperament and Character Inventory. *Compr Psychiatry* 2002; **43**(3)**:** 235-243.

6. Yang SS, Sung J, Kim J-H, Song Y-M, Lee K, Kim H-N *et al.* Some personality traits converse gradually by long-term partnership through the lifecourse: Genetic and environmental structure of Cloninger's temperament and character dimensions. *Journal of Psychiatric Research* 2015; **63:** 43-49.

7. Arnedo J, Svrakic DM, Del Val C, Romero-Zaliz R, Hernandez-Cuervo H, Fanous AH *et al.* Uncovering the hidden risk architecture of the schizophrenias: confirmation in three independent genome-wide association studies. *Am J Psychiatry* 2015; **172**(2)**:** 139-153.

8. Prado-Martinez J, Sudmant PH, Kidd JM, Li H, Kelley JL, Lorente-Galdos B *et al.* Great ape genetic diversity and population history. *Nature* 2013; **499**(7459)**:** 471-475.

9. Price AL, Patterson NJ, Plenge RM, Weinblatt ME, Shadick NA, Reich D. Principal components analysis corrects for stratification in genome-wide association studies. *Nat Genet* 2006; **38**(8)**:** 904-909.

10. Purcell S, Neale B, Todd-Brown K, Thomas L, Ferreira MA, Bender D *et al.* PLINK: a tool set for whole-genome association and population-based linkage analyses. *Am J Hum Genet* 2007; **81**(3)**:** 559-575.

11. Wu MC, Kraft P, Epstein MP, Taylor DM, Chanock SJ, Hunter DJ *et al.* Powerful SNP-set analysis for case-control genome-wide association studies. *Am J Hum Genet* 2010; **86**(6)**:** 929-942.

12. Wu MC, Lee S, Cai T, Li Y, Boehnke M, Lin X. Rare-variant association testing for sequencing data with the sequence kernel association test. *Am J Hum Genet* 2011; **89**(1)**:** 82-93.

13. Pirinen M, Donnelly P, Spencer CC. Including known covariates can reduce power to detect genetic effects in case-control studies. *Nat Genet* 2012; **44**(8)**:** 848-851.

14. Voorman A, Lumley T, McKnight B, Rice K. Behavior of QQ-plots and genomic control in studies of gene-environment interaction. *PLoS One* 2011; **6**(5)**:** e19416.

15. Schaid DJ, Rowland CM, Tines DE, Jacobson RM, Poland GA. Score tests for association between traits and haplotypes when linkage phase is ambiguous. *Am J Hum Genet* 2002; **70**(2)**:** 425-434.

16. Hunter DJ, Kraft P. Drinking from the fire hose--statistical issues in genomewide association studies. *N Engl J Med* 2007; **357**(5)**:** 436-439.

17. Liu D, Ghosh D, Lin X. Estimation and testing for the effect of a genetic pathway on a disease outcome using logistic kernel machine regression via logistic mixed models. *BMC Bioinformatics* 2008; **9:** 292.

18. Lin DY. An efficient Monte Carlo approach to assessing statistical significance in genomic studies. *Bioinformatics* 2005; **21**(6)**:** 781-787.

19. Kwee LC, Liu D, Lin X, Ghosh D, Epstein MP. A powerful and flexible multilocus association test for quantitative traits. *Am J Hum Genet* 2008; **82**(2)**:** 386-397.

20. Cheverud JM. A simple correction for multiple comparisons in interval mapping genome scans. *Heredity (Edinb)* 2001; **87**(Pt 1)**:** 52-58.

21. Nyholt DR. A simple correction for multiple testing for single-nucleotide polymorphisms in linkage disequilibrium with each other. *Am J Hum Genet* 2004; **74**(4)**:** 765-769.

22. Moskvina V, Schmidt KM. On multiple-testing correction in genome-wide association studies. *Genet Epidemiol* 2008; **32**(6)**:** 567-573.

23. Hoh J, Ott J. Mathematical multi-locus approaches to localizing complex human trait genes. *Nat Rev Genet* 2003; **4**(9)**:** 701-709.

24. Zaykin DV, Westfall PH, Young SS, Karnoub MA, Wagner MJ, Ehm MG. Testing association of statistically inferred haplotypes with discrete and continuous traits in samples of unrelated individuals. *Hum Hered* 2002; **53**(2)**:** 79-91.

25. Chapman JM, Cooper JD, Todd JA, Clayton DG. Detecting disease associations due to linkage disequilibrium using haplotype tags: a class of tests and the determinants of statistical power. *Hum Hered* 2003; **56**(1-3)**:** 18-31.

26. Schaid DJ, McDonnell SK, Hebbring SJ, Cunningham JM, Thibodeau SN. Nonparametric tests of association of multiple genes with human disease. *Am J Hum Genet* 2005; **76**(5)**:** 780-793.

27. Wessel J, Schork NJ. Generalized genomic distance-based regression methodology for multilocus association analysis. *Am J Hum Genet* 2006; **79**(5)**:** 792-806.

28. Mukhopadhyay I, Feingold E, Weeks DE, Thalamuthu A. Association tests using kernel-based measures of multi-locus genotype similarity between individuals. *Genet Epidemiol* 2010; **34**(3)**:** 213-221.

29. Tzeng JY, Zhang D. Haplotype-based association analysis via variance-components score test. *Am J Hum Genet* 2007; **81**(5)**:** 927-938.

30. Minichiello MJ, Durbin R. Mapping trait loci by use of inferred ancestral recombination graphs. *Am J Hum Genet* 2006; **79**(5)**:** 910-922.

31. Tachmazidou I, Verzilli CJ, De Iorio M. Genetic association mapping via evolution-based clustering of haplotypes. *PLoS Genet* 2007; **3**(7)**:** e111.

32. Zwir I, Shin D, Kato A, Nishino K, Latifi T, Solomon F *et al.* Dissecting the PhoP regulatory network of Escherichia coli and Salmonella enterica. *Proc Natl Acad Sci U S A* 2005; **102**(8)**:** 2862-2867.

33. Zwir I, Huang H, Groisman EA. Analysis of differentially-regulated genes within a regulatory network by GPS genome navigation. *Bioinformatics* 2005; **21**(22)**:** 4073-4083.

34. Schachtner R, Lutter D, Knollmuller P, Tome AM, Theis FJ, Schmitz G *et al.* Knowledge-based gene expression classification via matrix factorization. *Bioinformatics* 2008; **24**(15)**:** 1688-1697.

35. Sokal RR, Michener CD. A statistical method for evaluating systematic relationships. *University of Kansas Science Bulletin* 1958; **38:** 1409-1438.

36. Hartigan J, Wong M. Algorithm AS 136: A K-means clustering algorithm. *Applied Statistics* 1979**:** 100--108.

37. Pascual-Montano A, Carmona-Saez P, Chagoyen M, Tirado F, Carazo JM, Pascual-Marqui RD. bioNMF: a versatile tool for non-negative matrix factorization in biology. *BMC Bioinformatics* 2006; **7:** 366.

38. Harari O, Park SY, Huang H, Groisman EA, Zwir I. Defining the plasticity of transcription factor binding sites by Deconstructing DNA consensus sequences: the PhoP-binding sites among gamma/enterobacteria. *PLoS Comput Biol* 2010; **6**(7)**:** e1000862.

39. Tavazoie S, Hughes JD, Campbell MJ, Cho RJ, Church GM. Systematic determination of genetic network architecture. *Nat Genet* 1999; **22**(3)**:** 281-285.

40. Beer MA, Tavazoie S. Predicting gene expression from sequence. *Cell* 2004; **117**(2)**:** 185-198.

41. Ehret GB, Lamparter D, Hoggart CJ, Genetic Investigation of Anthropometric Traits C, Whittaker JC, Beckmann JS *et al.* A multi-SNP locus-association method reveals a substantial fraction of the missing heritability. *Am J Hum Genet* 2012; **91**(5)**:** 863-871.

42. Zwir I, Zaliz RR, Ruspini EH. Automated biological sequence description by genetic multiobjective generalized clustering. *Ann N Y Acad Sci* 2002; **980:** 65-82.

43. Cordon O, Herrera F, Zwir I. Linguistic modeling by hierarchical systems of linguistic rules. *Ieee Transactions on Fuzzy Systems* 2002; **10**(1)**:** 2-20.

44. Romero-Zaliz R, C. Rubio R, Cordón O, Cobb P, Herrera F, Zwir I. A multi-objective evolutionary conceptual clustering methodology for gene annotation within structural databases: a case of study on the gene ontology database. *IEEE Transactions on Evolutionary Computation*  2008; **12:6:** 679-701.

45. Arnedo J, Svrakic DM, del Val C, Romero‑Zaliz R, Hernández-Cuervo H, Molecular Genetics of Schizophrenia Consortium *et al.* Uncovering the Hidden Risk Architecture of the Schizophrenias: Confirmation in Three Independent Genome-‑Wide Association Studies. *The American journal of psychiatry* 2015; **172**(2)**:** 139-153.

46. Hinton GE, Salakhutdinov RR. Reducing the dimensionality of data with neural networks. *Science* 2006; **313**(5786)**:** 504-507.

47. Geiger JT, Weininger F, Gemmeke JF, Wollmer M, Schuller B, Rigoll G. Memory-enhanced neural networks and NMF for robust ASR. *IEEE/ACM Transactions on audio, speech, and language processing* 2014; **22**(6)**:** 1037-1046.

48. Le Roux J, Hershey JR, Weininger F. Deep NMF for speech separation. *IEEE Internatiional Conference on Acoustics, Speech, and Signal Processing (ICASSP)*. Mitsubishi Electric Research Laboratories, Inc: Cambridge, Massachusetts, 2015.

49. Cichocki A, Zdunek R, Phan AH, Amari S-i. *Nonnegative Matrix and Tensor Factorizations: Applications to Exploratory Multi-way Data Analysis and Blind Source Separation.* John Wiley & Sons, Inc2009.

50. Mejia-Roa E, Carmona-Saez P, Nogales R, Vicente C, Vazquez M, Yang XY *et al.* bioNMF: a web-based tool for nonnegative matrix factorization in biology. *Nucleic Acids Res* 2008; **36**(Web Server issue)**:** W523-528.

51. Lee DD, Seung HS. Learning the parts of objects by non-negative matrix factorization. *Nature* 1999; **401**(6755)**:** 788-791.

52. Tamayo P, Scanfeld D, Ebert BL, Gillette MA, Roberts CW, Mesirov JP. Metagene projection for cross-platform, cross-species characterization of global transcriptional states. *Proc Natl Acad Sci U S A* 2007; **104**(14)**:** 5959-5964.

53. Pascual-Montano A, Carazo JM, Kochi K, Lehmann D, Pascual-Marqui RD. Nonsmooth nonnegative matrix factorization (nsNMF). *IEEE transactions on pattern analysis and machine intelligence* 2006; **28:** 403-415.

54. Bezdek JC. Pattern Analysis. In: Pedrycz W, Bonissone PP, Ruspini EH (eds). *Handbook of Fuzzy Computation*. Institute of Physics Publishing Oxford University Press: Bristol, 1998, pp F6.1.1-F6.6.20.

55. Bezdek JC, Pal SK, IEEE Neural Networks Council. *Fuzzy models for pattern recognition : methods that search for structures in data*. IEEE Press: New York, 1992, xi, 539pp.

56. Brunet JP, Tamayo P, Golub TR, Mesirov JP. Metagenes and molecular pattern discovery using matrix factorization. *Proc Natl Acad Sci U S A* 2004; **101**(12)**:** 4164-4169.

57. Chagoyen M, Carmona-Saez P, Gil C, Carazo JM, Pascual-Montano A. A literature-based similarity metric for biological processes. *BMC Bioinformatics* 2006; **7:** 363.

58. Chagoyen M, Carmona-Saez P, Shatkay H, Carazo JM, Pascual-Montano A. Discovering semantic features in the literature: a foundation for building functional associations. *BMC Bioinformatics* 2006; **7:** 41.

59. Senbabaoglu Y, Michailidis G, Li JZ. Critical limitations of consensus clustering in class discovery. *Sci Rep* 2014; **4:** 6207.

60. Saeed F, Salim N, Abdo A. Voting-based consensus clustering for combining multiple clusterings of chemical structures. *J Cheminform* 2012; **4**(1)**:** 37.

61. Deb K. *Multi-objective optimization using evolutionary algorithms*. 1st edn. John Wiley & Sons: Chichester ; New York, 2001, xix, 497pp.

62. Deb K. Nonlinear goal programming using multi-objective genetic algorithms. *J Oper Res Soc* 2001; **52**(3)**:** 291-302.

63. Ruspini EH, Zwir I. Automated generation of qualitative representations of complex objects by hybrid soft-computing methods. In: Pal SK, Pal A (eds). *Pattern recognition : from classical to modern approaches*. World Scientific: New Jersey., 2002, pp 454-474.

64. Rissanen J. *Stochastic complexity in statistical inquiry*. World Scientific: Singapore, 1989, 177pp.

65. Romero-Zaliz R, Del Val C, Cobb JP, Zwir I. Onto-CC: a web server for identifying Gene Ontology conceptual clusters. *Nucleic Acids Res* 2008; **36**(Web Server issue)**:** W352-357.

66. Li DW, Wang C, Bruschweiler R. Maximal clique method for the automated analysis of NMR TOCSY spectra of complex mixtures. *J Biomol NMR* 2017; **68**(3)**:** 195-202.

67. Mofrad AA, Parker MG. Nested-Clique Network Model of Neural Associative Memory. *Neural Comput* 2017; **29**(6)**:** 1681-1695.

68. Rieck B, Fugacci U, Lukasczyk J, Leitte H. Clique Community Persistence: A Topological Visual Analysis Approach for Complex Networks. *IEEE Trans Vis Comput Graph* 2017.

69. Jiao Z, Ma K, Wang H, Zou L, Xiang J. Functional Connectivity Analysis of Brain Default Mode Networks Using Hamiltonian Path. *CNS Neurol Disord Drug Targets* 2017; **16**(1)**:** 44-50.

70. Goeman JJ, Buhlmann P. Analyzing gene expression data in terms of gene sets: methodological issues. *Bioinformatics* 2007; **23**(8)**:** 980-987.

71. Mitchell TM. *Machine Learning*. McGraw-Hill: New York, 1997, xvii, 414 p.pp.

72. Chawla NV, Bowyer KW, Hall LO, Kegelmeyer WP. SMOTE: Synthetic minority over-sampling technique. *J Artif Intell Res* 2002; **16:** 321-357.

73. Fernandez A, Garcia S, Herrera F, Chawla NV. SMOTE for Learning from Imbalanced Data: Progress and Challenges, Marking the 15-year Anniversary. *J Artif Intell Res* 2018; **61:** 863-905.

74. Hastie T, Tibshirani R, Friedman JH. *The elements of statistical learning : data mining, inference, and prediction : with 200 full-color illustrations*. Springer: New York, 2001, xvi, 533 p.pp.

75. Yu H, Luscombe NM, Lu HX, Zhu X, Xia Y, Han JD *et al.* Annotation transfer between genomes: protein-protein interologs and protein-DNA regulogs. *Genome Res* 2004; **14**(6)**:** 1107-1118.

76. A. Mehrle HR, I. Schupp, C. del Val, D. Arlt, F. Hahne, S. Bechtel, J. Simpson, O. Hofman, W. Hide, KH. Glatting, W. Huber, R. Pepperkok, A. Poustka, S. Wiemann. LIFEDB2006. *Nucleic Acid Research* 2006.

77. Previti C, Harari O, Zwir I, del Val C. Profile analysis and prediction of tissue-specific CpG island methylation classes. *BMC Bioinformatics* 2009; **10:** 116.

78. De Smet R, Marchal K. Advantages and limitations of current network inference methods. *Nat Rev Microbiol* 2010; **8**(10)**:** 717-729.

79. Zwir I, Latifi T, Perez JC, Huang H, Groisman EA. The promoter architectural landscape of the Salmonella PhoP regulon. *Mol Microbiol* 2012; **84**(3)**:** 463-485.

80. Zwir I, Yeo WS, Shin D, Latifi T, Huang H, Groisman EA. Bacterial nucleoid-associated protein uncouples transcription levels from transcription timing. *MBio* 2014; **5**(5)**:** e01485-01414.

81. Arnedo J, Romero-Zaliz R, Zwir I, Del Val C. A multiobjective method for robust identification of bacterial small non-coding RNAs. *Bioinformatics* 2014; **30**(20)**:** 2875-2882.

82. Ward LD, Kellis M. HaploReg: a resource for exploring chromatin states, conservation, and regulatory motif alterations within sets of genetically linked variants. *Nucleic Acids Res* 2012; **40**(Database issue)**:** D930-934.

83. Liu K, Yan Z, Li Y, Sun Z. Linc2GO: a human LincRNA function annotation resource based on ceRNA hypothesis. *Bioinformatics* 2013; **29**(17)**:** 2221-2222.

84. Chen X, Hao Y, Cui Y, Fan Z, He S, Luo J *et al.* LncVar: a database of genetic variation associated with long non-coding genes. *Bioinformatics* 2016.

85. Glazar P, Papavasileiou P, Rajewsky N. circBase: a database for circular RNAs. *RNA* 2014; **20**(11)**:** 1666-1670.

86. Li JH, Liu S, Zhou H, Qu LH, Yang JH. starBase v2.0: decoding miRNA-ceRNA, miRNA-ncRNA and protein-RNA interaction networks from large-scale CLIP-Seq data. *Nucleic Acids Res* 2014; **42**(Database issue)**:** D92-97.

87. Huang da W, Sherman BT, Lempicki RA. Systematic and integrative analysis of large gene lists using DAVID bioinformatics resources. *Nat Protoc* 2009; **4**(1)**:** 44-57.

88. Du J, Yuan Z, Ma Z, Song J, Xie X, Chen Y. KEGG-PATH: Kyoto encyclopedia of genes and genomes-based pathway analysis using a path analysis model. *Mol Biosyst* 2014; **10**(9)**:** 2441-2447.

89. Karp PD, Billington R, Holland TA, Kothari A, Krummenacker M, Weaver D *et al.* Computational Metabolomics Operations at BioCyc.org. *Metabolites* 2015; **5**(2)**:** 291-310.

90. Kupershmidt I, Su QJ, Grewal A, Sundaresh S, Halperin I, Flynn J *et al.* Ontology-based meta-analysis of global collections of high-throughput public data. *PLoS One* 2010; **5**(9).

91. Lango Allen H, Estrada K, Lettre G, Berndt SI, Weedon MN, Rivadeneira F *et al.* Hundreds of variants clustered in genomic loci and biological pathways affect human height. *Nature* 2010; **467**(7317)**:** 832-838.

92. Purcell SM, Wray NR, Stone JL, Visscher PM, O'Donovan MC, Sullivan PF *et al.* Common polygenic variation contributes to risk of schizophrenia and bipolar disorder. *Nature* 2009; **460**(7256)**:** 748-752.

93. Jaffe AE, Storey JD, Ji H, Leek JT. Gene set bagging for estimating the probability a statistically significant result will replicate. *BMC Bioinformatics* 2013; **14:** 360.

94. Lu Q, Yao X, Hu Y, Zhao H. GenoWAP: GWAS signal prioritization through integrated analysis of genomic functional annotation. *Bioinformatics* 2016; **32**(4)**:** 542-548.

95. Stone JL, Merriman B, Cantor RM, Geschwind DH, Nelson SF. High density SNP association study of a major autism linkage region on chromosome 17. *Hum Mol Genet* 2007; **16**(6)**:** 704-715.

96. Arnedo J, Mamah D, Baranger DA, Harms MP, Barch DM, Svrakic DM *et al.* Decomposition of brain diffusion imaging data uncovers latent schizophrenias with distinct patterns of white matter anisotropy. *Neuroimage* 2015; **120:** 43-54.

**C. Supplementary Tables**

**Table S1**. Descriptors for high and low scorers on TCI subscales.

**Table S2**. Calculation of the each subject's level on previously validated indicators of ill-being and well-being. Ill-being is indicated by low scores on SD + CO. Well-being is the product SD x CO x ST. The Boolean risk of ill-being is the bottom decile of its indicator for 1 and 0 otherwise. The Boolean classification of well-being is the top decile of its indicator for 1 and 0 otherwise. Semi-continuous ratings from 1 to 6 are also given by separating subjects into 6 equal classes based on their normalized (percentile) rank from 0 to 1.

**Table S3**. Molecular consequences and chromosomal characterization of the SNPs identified within each SNP Set.

**Table S4**. List of SNP sets associated with character sets and their assigned names based on differentiating pathways/processes. SNP sets are grouped by the character profile with which they are most frequently associated to facilitate recognition of the functional differences between the groups.

**Table S5**. Description of the different types and subtypes of genes based on their molecular features and location. Ensembl descriptions of the molecular consequences of different types of variants are also summarized.

**Table S6**. Summary of 727 Genes identified as components of the 42 SNP sets significantly associated with Character Sets and Profiles. The genes are identified by their ENSEMBL code and gene name. Their chromosomal assignment, known functions and gene type are shown. The columns to the right show the genes per SNP set, with presence of a gene in a particular SNP set indicated by Yes in the corresponding box for the intersection of the gene and the SNP set.

**Table S7**. Pathways associated with Character profiles. The number of genes associated with any of the character profiles and with particular profiles is shown (Source KEGG and DAVID pathways databases). Differences among profiles were evaluated by ANOVA (p-value<146E-19) and T-test (p-value<0.05, Bonferroni) for comparisons between pairs of profiles. Apathetic differ from Creative, Resourceful; Dependent differs from Organized, Creative and Resourceful; Organized differs from Dependent, Creative and Resourceful; Creative differs from Apathetic, Dependent and Organized; Resourceful differs from Apathetic, Dependent and Organized

**Table S8**. Summary of the genes present in each of the 5 character profiles and shared by particular pairs of profiles (i.e., intersection of profiles).

The Totals refer to the total number of genes mapped to each of the 5 profiles. Odd-diagonal cells display the number of genes in the intersection of particular pairs of profiles. The cells in the diagonal exhibit the number of genes unique for a particular profile (i.e., not present in any other). Percentage calculations are based on the total 727 genes to facilitate comparisons.

**Table S9**. (A) GO Molecular Processes corresponding to each Pipeline that flows to a particular Character Scale. The component profiles of the pipeline are shown for each of the three TCI character traits (SD, CO, or ST). Then the total number of genes associated with each component of the pipeline is shown. The count of the number of genes associated with a particular GO pathway is given along with its percentage of the total. In the rightmost column the significance of the association of the GO pathway as a contributor to that component of the pipeline is given as the p-value based on Hypergeometric (Fisher's exact test).

(B) GO Molecular Processes contributing to overall Self-directedness scale values. Contributions from each component of the pipeline are ordered by number of genes contributed by each component beginning with the component with the greatest number of genes. Differences between components in the number of contributing genes were significant when tested by ANOVA (p-value<1.99E-15. Disjoint MP are shown for each profile, but only Organized is significantly different from the others (T-test p-value<0.05, Bonferroni).

(C) GO Molecular Processes contributing to overall Cooperativeness scale values. Contributions from each component of the pipeline are ordered by number of genes contributed by each component beginning with the component with the greatest number of genes. Differences between components in the number of contributing genes were significant when tested by ANOVA (p-value<2.74E-13). Disjoint Molecular Processes are shown for each profile, but only Organized is significantly different (T-test p-value<0.05, Bonferroni).

(D) GO Molecular Processes contributing to overall Self-transcendence scale values. Contributions from each component of the pipeline are ordered by number of genes contributed by each component beginning with the component with the greatest number of genes. Differences between components in the number of contributing genes were significant when tested by ANOVA (p-value<6.00E-3). Disjoint Molecular Processes are shown for each profile, but only genes associated with both the Creative and Organized profiles are significantly different from the other components (T-test p-value<0.05, Bonferroni).

**Table S10**. Description of the known features of the genes in the pipeline that contribute to TCI Self-directedness. Gene descriptions from different sources are displayed. From left to right, genes within Creative (Only), Organized (Only), Resourceful, Organized & Dependent (Only), and Organized & Apathetic (Only) profiles are described. Each gene is identified by ENSEMBL code and name, chromosome location, and known functions, molecular processes, and medical complications.

**Table S11**. Description of the known features of the genes in the pipeline that contribute to TCI Cooperativeness. Gene descriptions from different sources are displayed. From left to right, genes within Creative (Only), Organized (Only), Dependent (Only), Organized & Dependent (only), and Organized & Apathetic (Only) profiles are described. Each gene is identified by ENSEMBL code and name, chromosome location, and its known functions, molecular processes, and medical complications.

**Table S12**. Description of the known features of the genes in the pipeline that contribute to TCI Self-transcendence. Gene descriptions from different sources are displayed. From left to right, genes within Creative and Apathetic (All), Creative and Organized (All), and Creative (Only) are described. Each gene is identified by ENSEMBL code and name, chromosome location, and its known functions, molecular processes, and medical complications.

**Table S13**. Switching genes from different states: well, ill, and intermediate. Genes are grouped by their corresponding SNP sets in Bold.

**Table S14**. Groups of genes associated with TCI character in Finnish sample that are regulatory targets of 276 particular miRNAs identified in the TRANSFAC database. The strength of the association of character-related genes with particular miRNAs is indicated by the p-value based on Fisher's exact test (Hypergeometric statistics).

**Table S15**. SNP Sets identified by PGMRA (p-value <1E-05) in the Finnish sample and replicated in the Korean (91%) and German (86%) samples. The replication score was calculated using Hypergeometric statistics.

**Table S16**. Character Sets identified by PGMRA in the Finnish sample and replicated in the Korean (93%) and German (82%) samples. The replication score was calculated using Hypergeometric statistics.

**Table S17**. Genotypic-phenotypic Relationships among SNP and Character Sets identified in the Finnish sample and replicated in the Korean (94%) and German (84%) samples. The replication score was calculated using Hypergeometric statistics and Multi-objective optimization techniques (see Pareto values in Tables S18 and S19)

**Table S18**. Match between Genotypic-Phenotypic Relationships between the Finnish and Korean samples. The replication score was calculated using Hypergeometric statistics. Scores include the match between SNP Sets and Character Sets in both samples, as well as that of the Genotypic-Phenotypic Relationships in the Korean sample. Non-dominated solutions among these three measurements were selected as the Pareto optimal frontier (see Supplementary Methods).

**Table S19**. Match between Genotypic-Phenotypic Relationships between the Finnish and German samples. The replication score was calculated using Hypergeometric statistics. Scores include the match between SNP Sets and Character Sets in both samples, as well as that of the Genotypic-Phenotypic Relationships in the German sample. Non-dominated solutions among these three measurements were selected as the Pareto optimal frontier (see Supplementary Methods).

**Table S20**. Genes uncovered in this study that were previously reported in association TCI personality traits (temperament and/or character).

**Table S21**. Summary of genes uncovered in this study that were previously reported in association with TCI Character measures. (Rectangles contain same genes with different names, ‘? ‘ indicates a relationship with TCI, and ‘YES’ exhibits a specific relationship with a Character scale.)

**Table S22**. Analysis of the variance in the 3 samples. Raw data indicates the SNPs recognized by the SNP sets identified by PGMRA (p-value <1E-05) associated with the Character phenotype (Table 1), and those from their homologs (best match only) in the other samples (Table S15). Outliers were identified as described in Methods.

**Table 23**. Environmental analysis. (A) Definition of environmental variables. (B) Direct associations of Character sets and Environmental sets (C) Direct associations of SNP sets and Environmental sets (D) Indirect associations between SNP sets and Character sets dependent on Environmental sets as mediators (i.e., proxy)

**D. Supplementary Figures**

**Figure S1**. Schematic of the PGMRA method applied to multiple domains of knowledge (see Supplementary Method). (A) Deep learning network developed by PGMRA fusing different domain of knowledge in a semi-supervised fashion including: unsupervised autoencoding, multiobjective optimization and pooling, interpretable association of types of knowledge, labeling the associations, and developing a classifier. Each layer has its own learning process and constitutes the input of the next layer. (B) Schematic of the genotypic-phenotypic associations identified by PGMRA in an unsupervised fashion. (C) Flow chart of the PGMRA process. (D) PGMRA performs a deep unsupervised NMF learning process: (i) NMF is implemented based on decomposing an input dataset, encoded as a matrix (or a tensor) composed of features and observations/subjects, into smaller factors. The learning process is a mirror process because it consists of comparing the original matrix with that reconstructed from the factors, and adjust those factors by the error. Factors are derived by combining matrix W and H. (iii) NMF can be transformed into a supervised method by moving matrix W to the other side of the equation applying the pseudoinverse of a product. (iii) Illustration of the process carried out by the NMF method to learn one factor: ordering the columns of W, as well as the rows of H, and multiply them. (E) Deep NMF process systematically applied (convolutive) using different number of maximum clusters or granularity levels (Consensus clustering). Optimal submatrices (factors) are selected from all levels by a multiobjective optimization process. This image can also illustrate the recurrent application of NMF to identify high level profiles. (F) Schematic that exemplifies how PGMRA sees the biomedical datasets (GWAS, DTI images, etc.). 6 patients have a deficit (value = 20) in different regions. Typically, the average of each cell is calculated, and, as a consequence, there is no region with a particular deficit (all values = 87) in all patients. Because averaging the cells conceals the differences among patients, PGMRA is focused on segmentation of patients and features .

**Figure S2**. Histogram representing the chromosomal location of genes corresponding to SNP Sets related to Character Sets. The bars revealed differences between genes related only to Character Sets (red), to Character and Temperament Sets (blue color), and all genes (green color). (Temperament Sets are shown elsewhere.)

**Figure S3**. Dissection of a GWAS in a Finnish population and identification of the Genotypic and Phenotypic architecture of personality measured by the TCI. Genotypic network, where nodes indicate SNP Sets linked by shared SNPs (blue line) and/or subjects (red line). Five character profiles are distinguished: Resourceful, Organized, Creative, Dependent, and Apathetic. (A) Subnetwork involving SNP Sets that only match to Character Sets (Dashed border) unrelated to Temperament sets (Temperament Sets are shown elsewhere). (B) Health status (ill, well, intermediate) is color-coded and superimposed onto the genotypic-phenotypic architecture of personality displayed in (A).

**Figure S4**. Surface representing the ill-being status of the uncovered SNP Sets. The ill-being status (z-axis; red high; green: low) was calculated based on the distribution of the status of subjects (Table S2) within each SNP Set, and the surface was plotted interpolating the relation domains. The order adopted for plotting SNP Sets is calculated based on clustering shared subjects (x-axis) and shared SNPs (y-axis) using Hypergeometric statistics (see Supplementary Method). (Close-located SNP Sets in an edge share more SNPs and/or subjects than those located far away.).

**Figure S5**. Surface representing the ill-being status of the uncovered Genotypic-phenotypic relationships between Character and SNP Sets. These values (z-axis; red high; green: low) were calculated based on the distribution of the status of subjects (Table S2) within each relationship, and the surface was plotted interpolating the relation domains. The order adopted for plotting SNP Sets is calculated based on clustering shared subjects in SNP (x-axis) and in Character (y-axis) Sets using Hypergeometric statistics (see Supplementary Method). (Close-located SNP or Character Sets in an edge share more subjects than those located far away.)

**Figure S6**. Evaluation of well- and ill-being measurements in Character Sets and their relationships with SNP Sets using Anova statistics. (A) Ill-being and (B) well-being measurements were compared for the different character profiles.

**Figure S7**. The German sample was screened to exclude psychopathology, including personality disorders, so it had a reduced number of individuals with unhealthy personalities, as it is seen by the distribution/histogram of well-being in the Finnish and German Samples. First, we estimated the 3rd quantile in the Finnish normal distribution that suggests a value of 0.71 (SD+CO/Max in the unit interval). Second, we calculated the proportion of the Finnish that are equal or higher than this value: 24%. Third, we calculated the number of Germans that are above that reference value: 89%. Fourth, we calculated the Wilcoxon rank test from these two populations >0.71 and the p-value is 1.13E-81.

**Figure S8.** QQ Plot with adjustment by sex and 3 PCs as covariates (red color) and without covariates (black color).

**Figure S9**. AUC analysis of the TCI measurements. (A) AUC calculated based on the Character dichotomous Well-being measurement on individual 5-fold Cross-validation, and (B) the average of these folds. (C) AUC calculated based on the Character dichotomous ill-being measurement on individual 5-fold Cross-validation, and (D) the average of these folds.
