# Supplementary figures and images for: Uncovering the complex genetics of human character
Source: Mol Psychiatry. 2018 Oct 3;25(10):2295–312. doi: 10.1038/s41380-018-0263-6 (PMC7515844; doi:10.1038/s41380-018-0263-6)

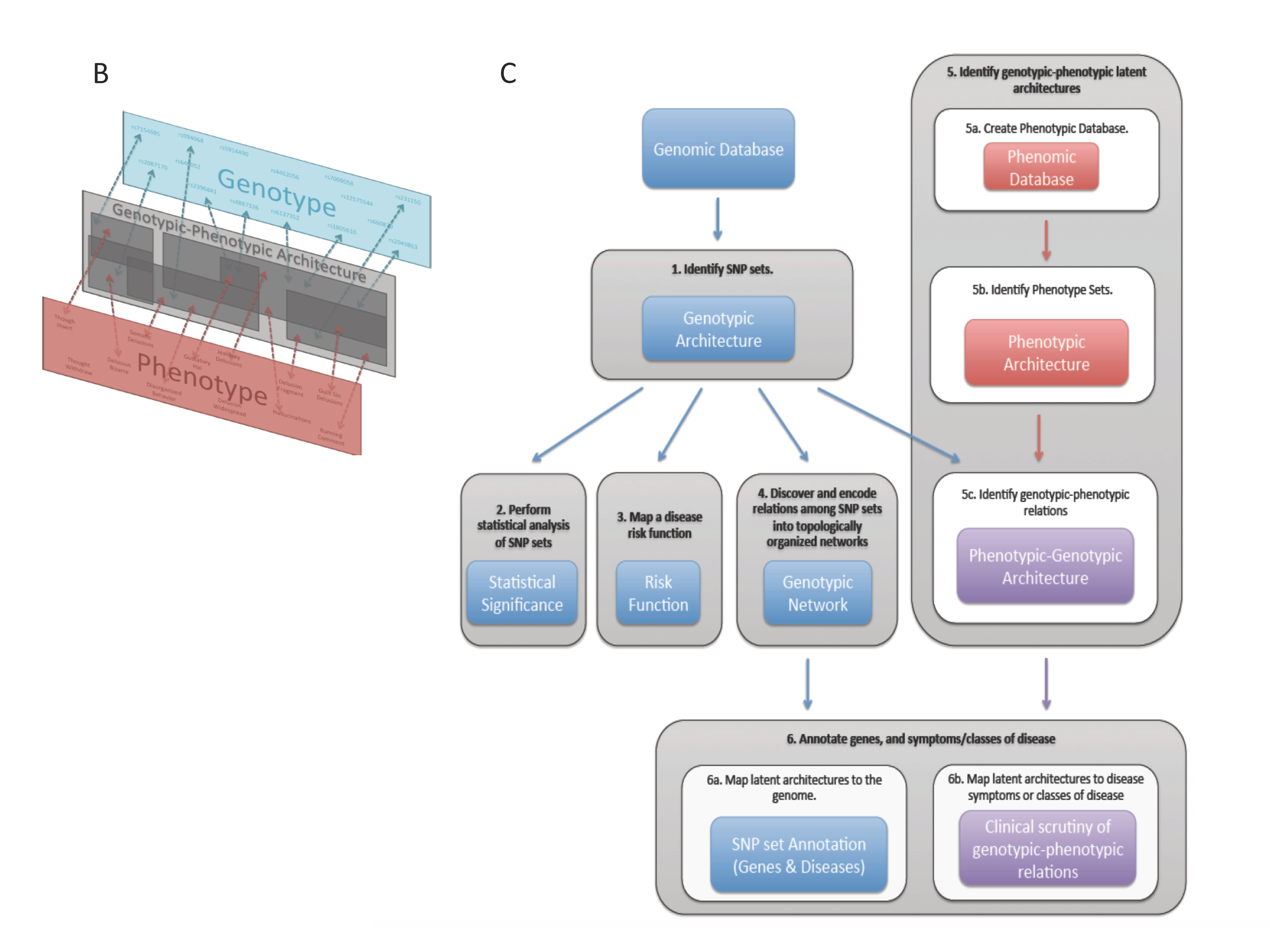

Supplement: Supplementary file 4 — Figure S1B-C [file 41380_2018_263_MOESM4_ESM.tif]

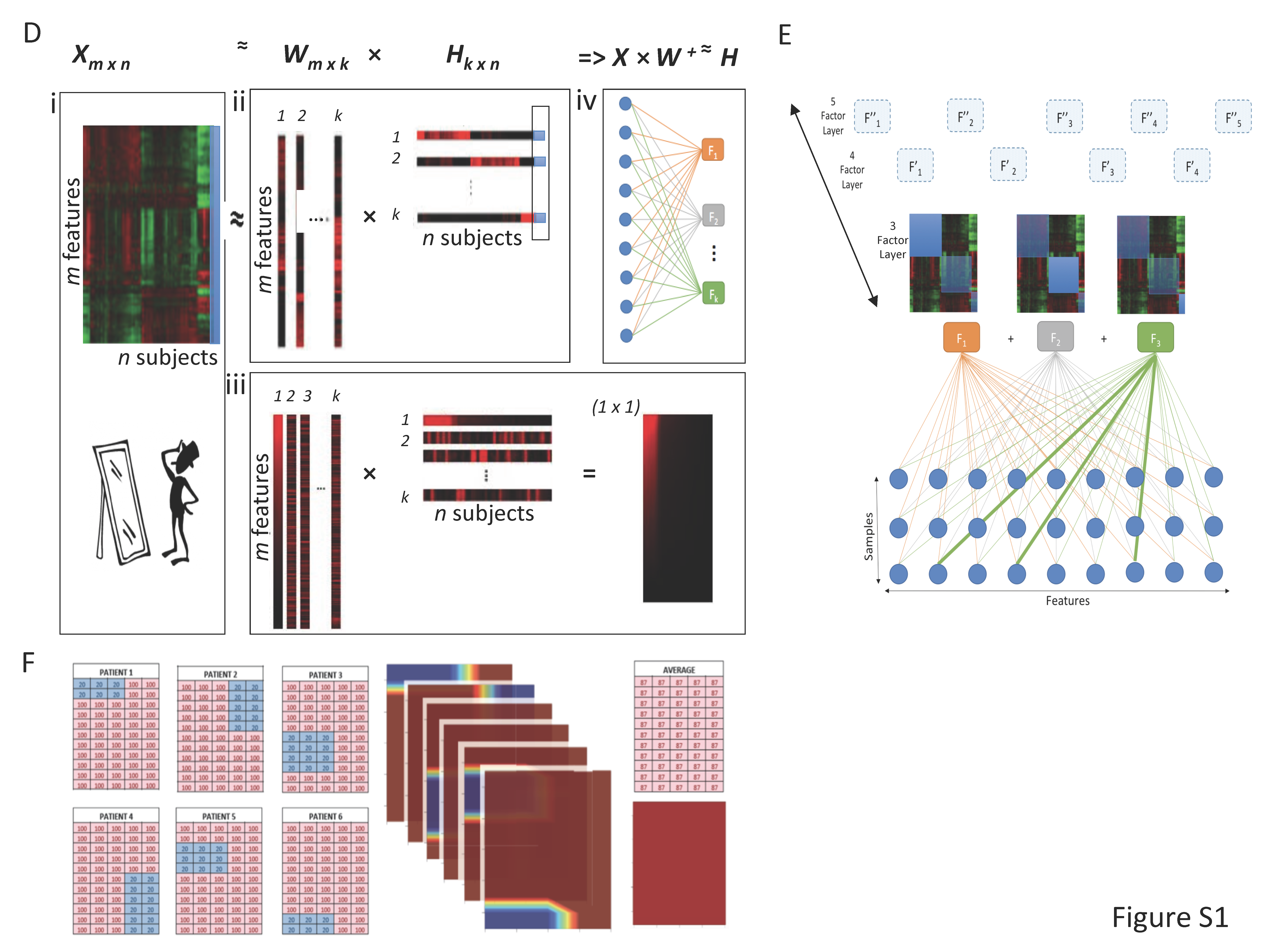

Supplement: Supplementary file 5 — Figure S1D-F [file 41380_2018_263_MOESM5_ESM.tif]

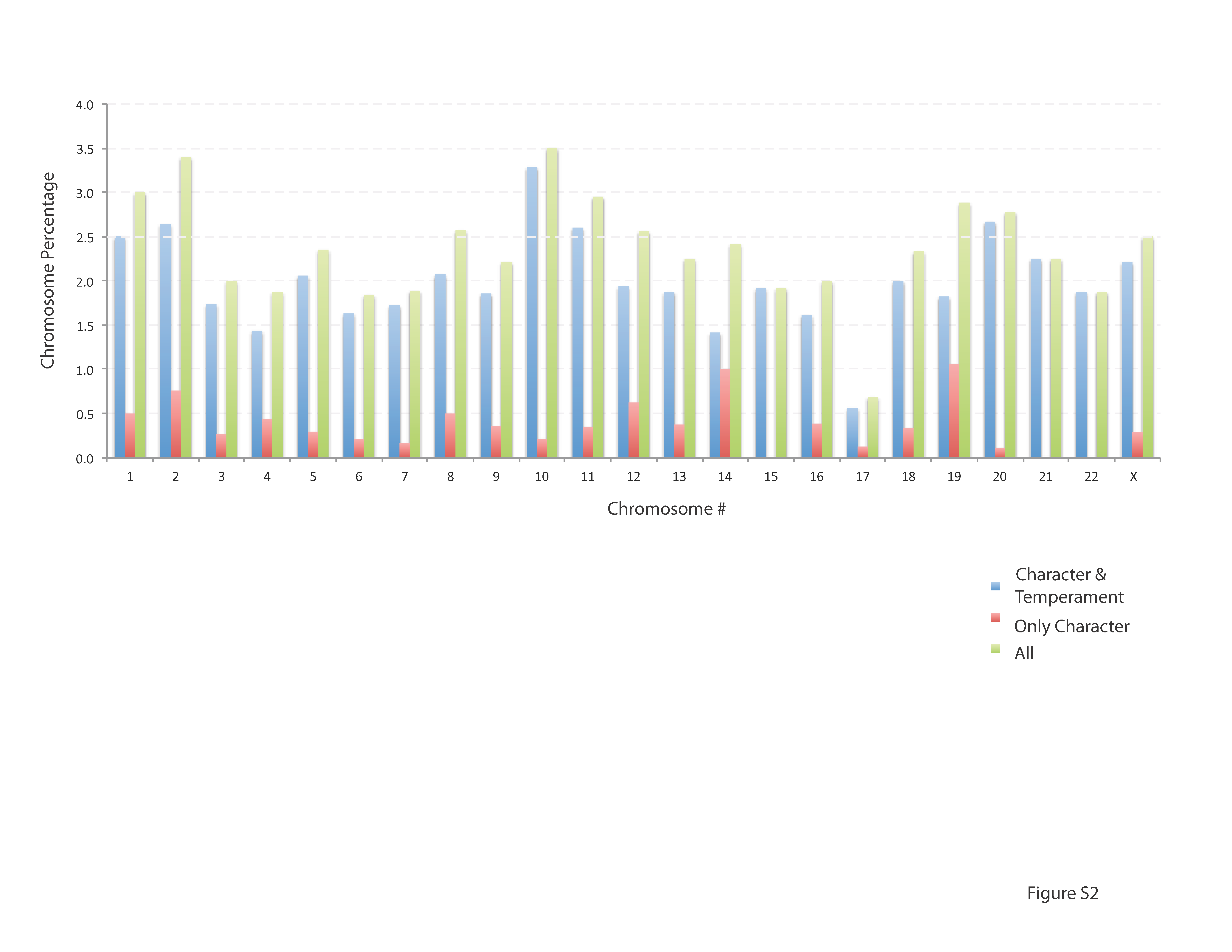

Supplement: Supplementary file 6 — Figure S2 [file 41380_2018_263_MOESM6_ESM.tif]

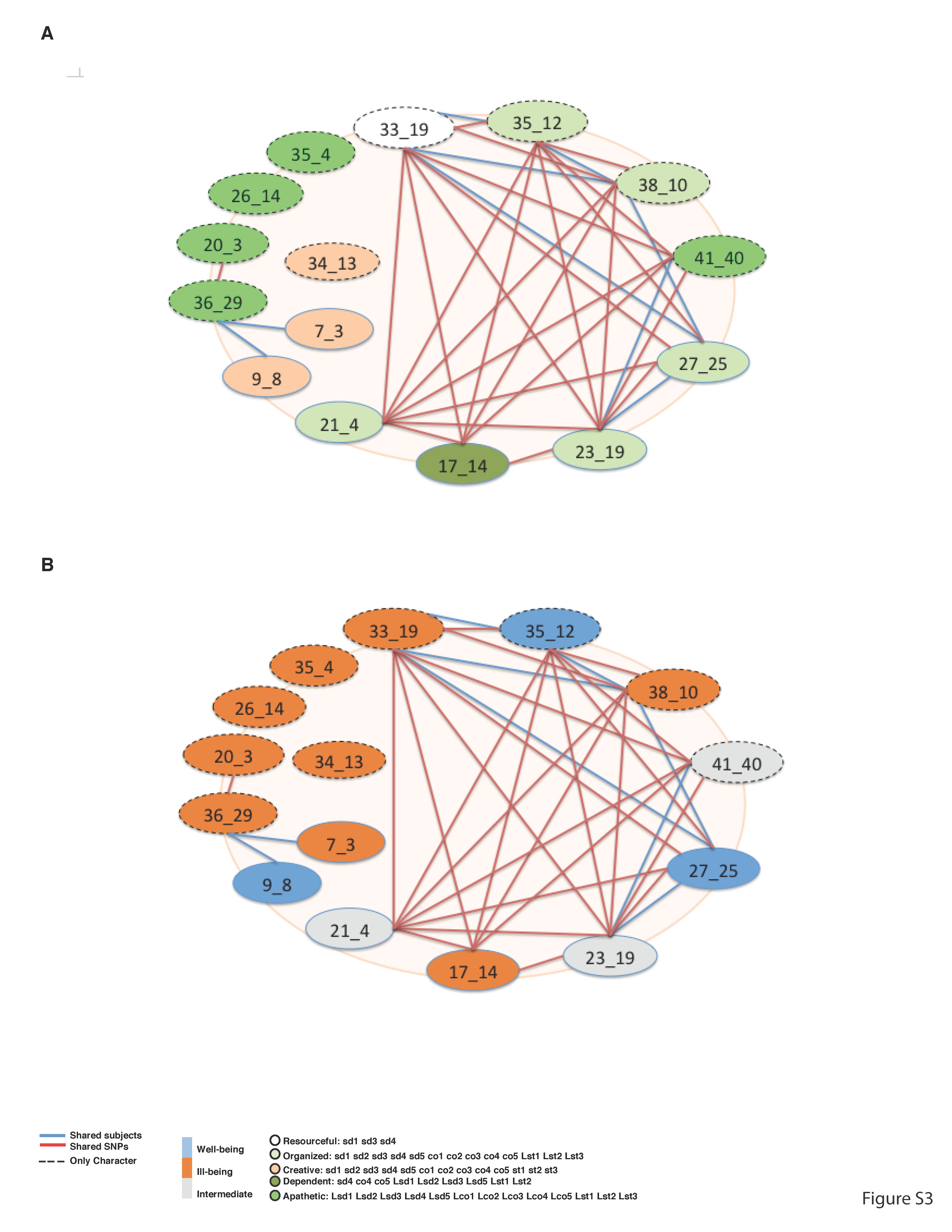

Supplement: Supplementary file 7 — Figure S3 [file 41380_2018_263_MOESM7_ESM.tif]

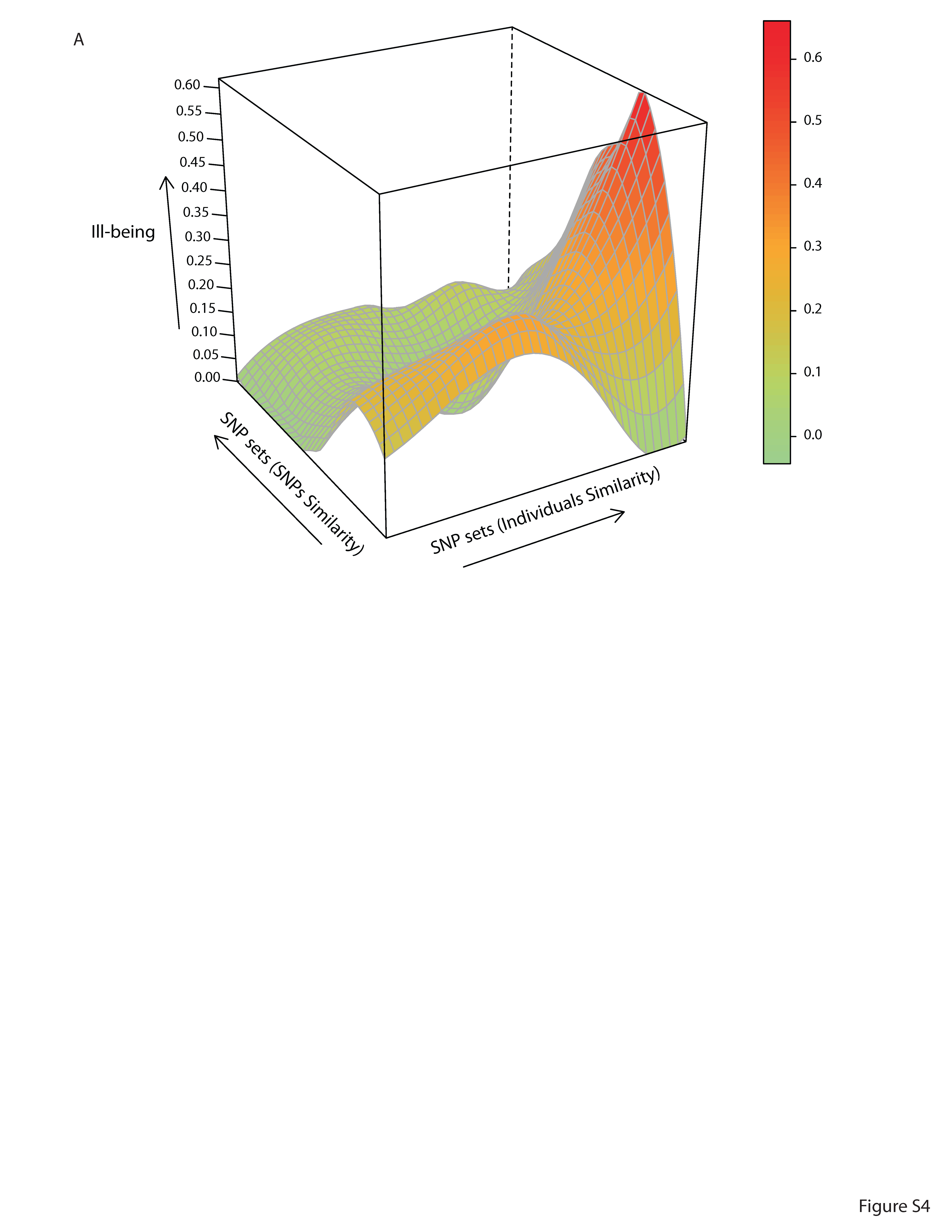

Supplement: Supplementary file 8 — Figure S4 [file 41380_2018_263_MOESM8_ESM.tif]

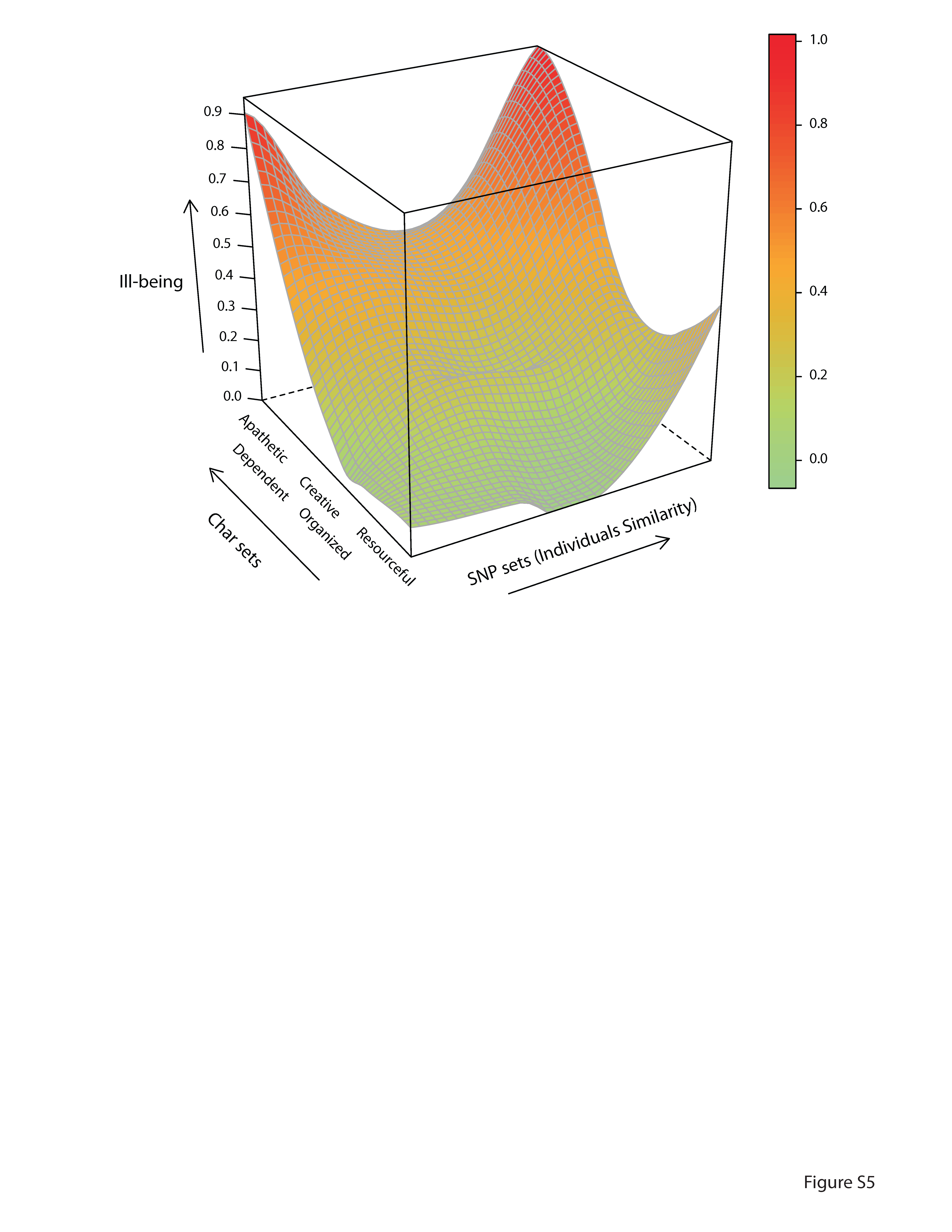

Supplement: Supplementary file 9 — Figure S5 [file 41380_2018_263_MOESM9_ESM.tif]

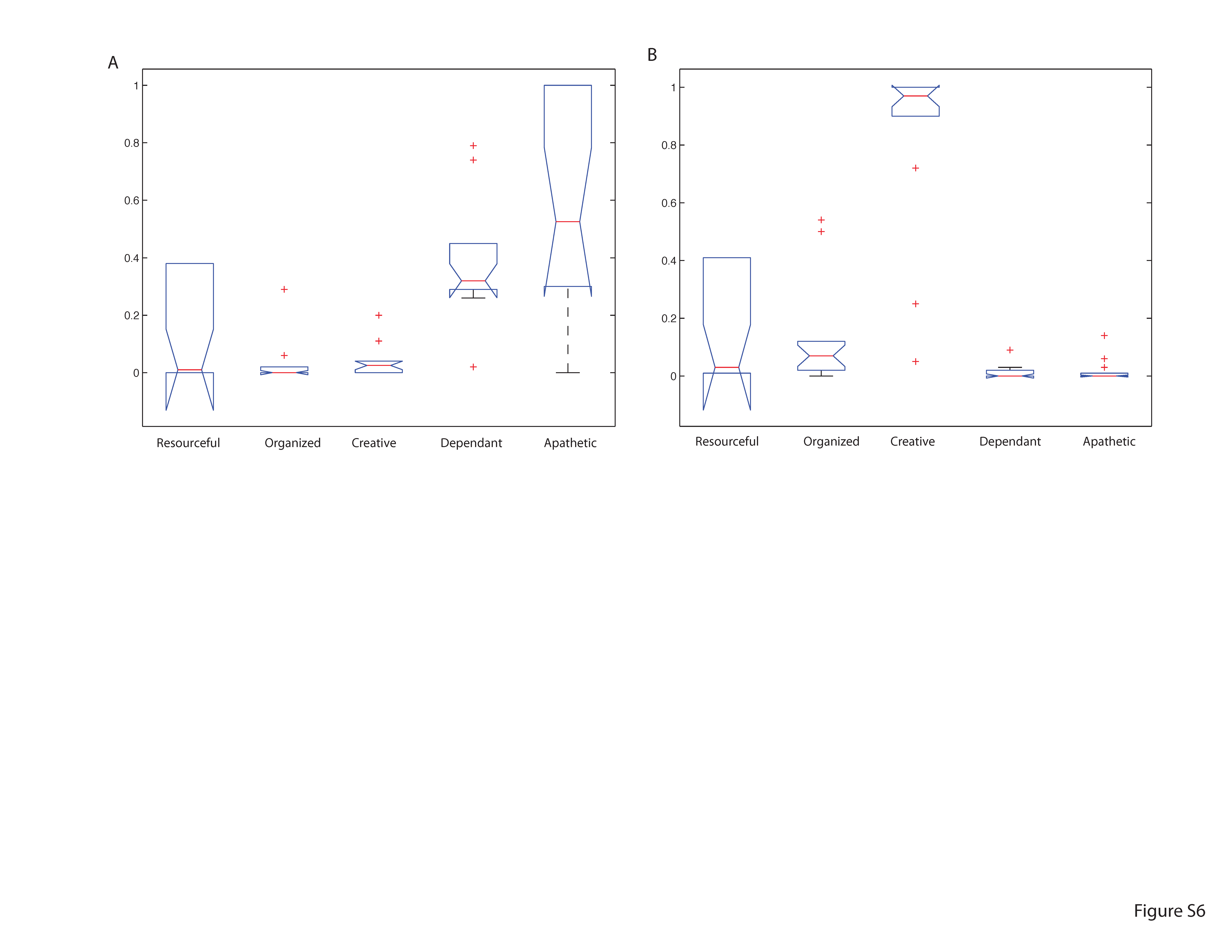

Supplement: Supplementary file 10 — Figure S6 [file 41380_2018_263_MOESM10_ESM.tif]

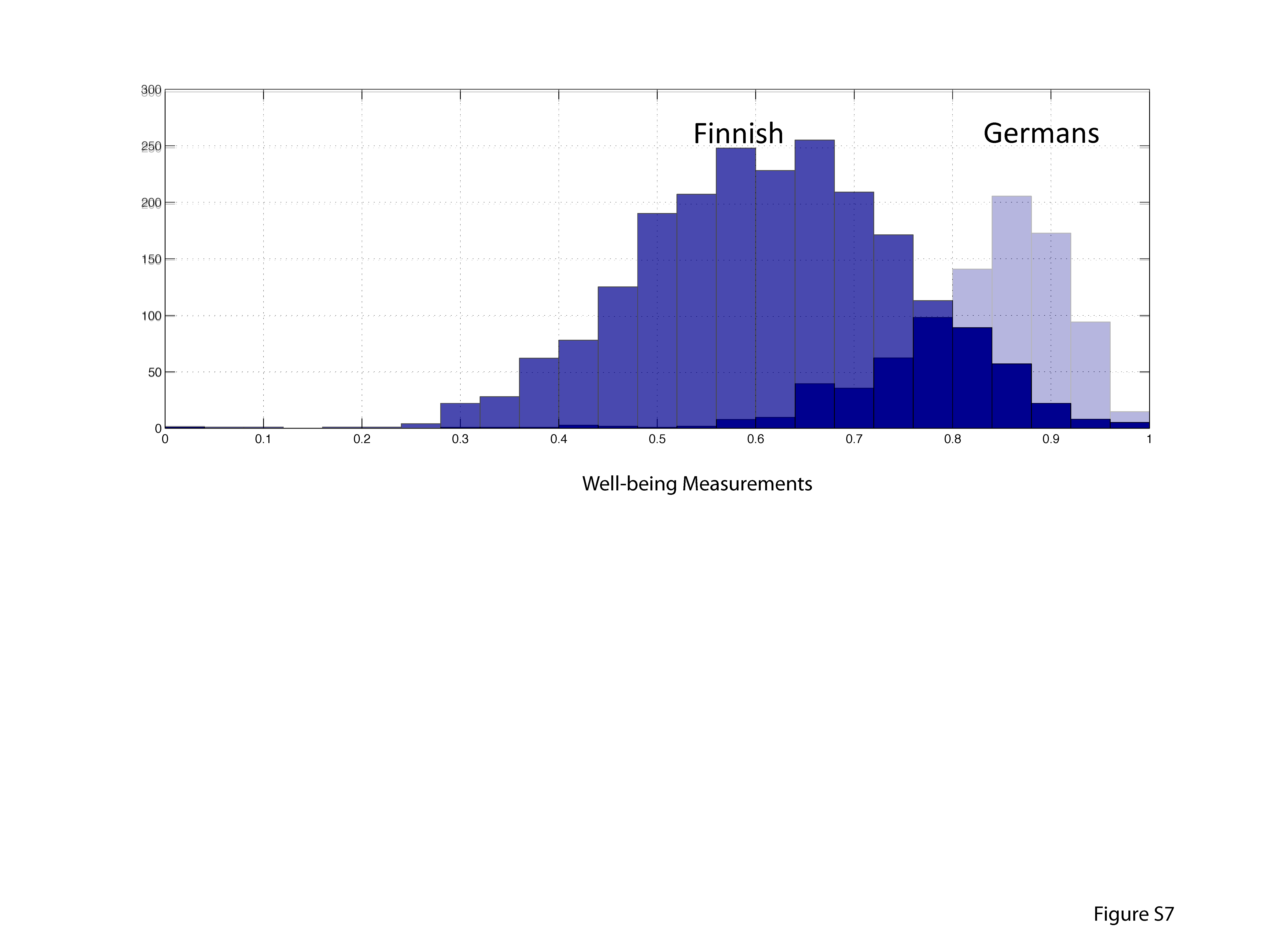

Supplement: Supplementary file 11 — Figure S7 [file 41380_2018_263_MOESM11_ESM.tif]
